# A Conserved DNA Repeat Promotes Selection of a Diverse Repertoire of *Trypanosoma brucei* Surface Antigens from the Genomic Archive

**DOI:** 10.1371/journal.pgen.1005994

**Published:** 2016-05-05

**Authors:** Galadriel Hovel-Miner, Monica R. Mugnier, Benjamin Goldwater, George A. M. Cross, F. Nina Papavasiliou

**Affiliations:** 1 The Rockefeller University, Laboratory of Lymphocyte Biology, New York, New York, United States of America; 2 The George Washington University, Department of Microbiology Immunology, and Tropical Medicine, Washington, DC, United States of America; 3 The Rockefeller University, Laboratory of Molecular Parasitology, New York, New York, United States of America; Fred Hutchinson Cancer Research Center, UNITED STATES

## Abstract

African trypanosomes are mammalian pathogens that must regularly change their protein coat to survive in the host bloodstream. Chronic trypanosome infections are potentiated by their ability to access a deep genomic repertoire of Variant Surface Glycoprotein (*VSG*) genes and switch from the expression of one *VSG* to another. Switching *VSG* expression is largely based in DNA recombination events that result in chromosome translocations between an acceptor site, which houses the actively transcribed *VSG*, and a donor gene, drawn from an archive of more than 2,000 silent *VSG*s. One element implicated in these duplicative gene conversion events is a DNA repeat of approximately 70 bp that is found in long regions within each BES and short iterations proximal to *VSG*s within the silent archive. Early observations showing that 70-bp repeats can be recombination boundaries during *VSG* switching led to the prediction that *VSG*-proximal 70-bp repeats provide recombinatorial homology. Yet, this long held assumption had not been tested and no specific function for the conserved 70-bp repeats had been demonstrated. In the present study, the 70-bp repeats were genetically manipulated under conditions that induce gene conversion. In this manner, we demonstrated that 70-bp repeats promote access to archival *VSG*s. Synthetic repeat DNA sequences were then employed to identify the length, sequence, and directionality of repeat regions required for this activity. In addition, manipulation of the 70-bp repeats allowed us to observe a link between *VSG* switching and the cell cycle that had not been appreciated. Together these data provide definitive support for the long-standing hypothesis that 70-bp repeats provide recombinatorial homology during switching. Yet, the fact that silent archival *VSGs* are selected under these conditions suggests the 70-bp repeats also direct DNA pairing and recombination machinery away from the closest homologs (silent BESs) and toward the rest of the archive.

## Author Summary

Chromosomal translocations can fuel genetic change or cause catastrophic genomic damage. African trypanosomes, exemplified by *Trypanosoma brucei* sub-species, are unicellular parasites that can chronically infect their human and livestock hosts by using a strategy of antigenic variation by which they repeatedly change their protein coats. Switching the surface coat requires the accurate selection and translocation of a single silent coat gene, from a large genomic archive, into an actively transcribed site. How the coat genes from within this deep archive are selected and activated was unproven. Here we show that a specific repetitive DNA sequence is required to access coat genes from diverse sites within the genome. The likely outcome of restricting this process of coat gene selection in natural infections would be a reduction in the chronic nature of African trypanosomiasis.

## Introduction

African trypanosomes are protozoan parasites that have dedicated more than 20% of their coding capacity [1,2] and 10% total cellular protein content [3] to a single biological function. To survive in the challenging environmental niche of the mammalian bloodstream, subspecies of *Trypanosoma brucei* must regularly change their antigenic glycoprotein coat. In this manner, they are able to escape the antibody-mediated immune response of their host to cause a chronic infection of the bloodstream that results in death of both humans (African sleeping sickness) and livestock (nagana) if left untreated [4]. Each parasite’s coat is composed of a densely packed single member of a large family of Variant Surface Glycoproteins (VSG) [5], which are thought to share a conserved membrane-bound structure but are encoded by highly divergent genes [2].

The *T*. *brucei* genome encodes more than 2000 *VSG* genes and *VSG* pseudogenes within a genome consisting of 11 megabase chromosomes (MBC), a variable number (usually 5–10) of intermediate chromosomes, and about 100 minichromosomes (MC) [2,6]. Yet, only one *VSG* is expressed at a given time from one of ~15 possible Bloodstream Expression Sites (BES) located at the subtelomeres of MBCs [7]. BESs share a similar sequence and organization, including an RNA polymerase I promoter, a series of Expression Site Associated Genes (ESAGs), a large region of repetitive DNA (70-bp repeats) that precede *VSG* gene, which is located a short distance upstream of telomere [7]. While minichromosomal *VSGs* are also subtelomeric, the majority of the *VSG* archive is located in *VSG* arrays on the arms of the MBCs [1]. Survival of *T*. *brucei* in the bloodstream requires the regular activation of silent *VSGs* from the genomic archive.

Switching from the expression of one *VSG* coat to the next predominantly occurs by three genetic mechanisms. A change in the BES being transcribed, resulting in the expression of its subtelomeric *VSG*, is termed In Situ (IS) switching [8]. Telomeric Exchange (TE) is homologous recombination between subtelomeres that results in the exchange of a silent *VSG* with one in the active BES, retaining both *VSG* genes [9]. In contrast, duplicative Gene Conversion (GC), as the name implies, results in the duplication of a silent *VSG* donor into the active BES and simultaneous deletion of the previously expressed *VSG* gene [10]. Unlike IS and TE, which activate silent *VSGs* already located at subtelomeric sites, GC is the mechanism of *VSG* switching that permits access to the entire *VSG* archive (BES, MC, and MBC arrays). GC is thought to be the predominant mechanism during natural infections [11] and can be activated under laboratory conditions, where rates of switching are low (~1x10^-5^), by increasing subtelomeric DNA breakage at the active BES [12–14]. Among all switching mechanisms there appears to be a semi-predictable hierarchy of *VSG* gene selection that begins with the selection of BES-encoded subtelomeric *VSGs*, followed by non-BES subtelomeric *VSGs* (such as those on MCs), and finally those from non-telomeric sites in the genome (loosely organized *VSG* arrays) [15]. Selection of *VSGs* from other BESs is highly favored during early switch events and is the most common gene selection preference observed under laboratory conditions [12,15]. This is probably because BESs have very similar DNA sequences, including regions of near identity for many kilobases, which would provide ample homology for recombination during gene conversion [7]. Selection of BES-encoded *VSGs* alone, of which there are about 15, would not be expected to support chronic *T*. *brucei* infection.

DNA repeat expansions are a common source of genomic translocations (like gene conversions) and genomic instability among eukaryotic genomes, and can result in genetic disorders in humans (reviewed in [16]). Thus, the discovery that the 5’ limit of translocation during *VSG* switching was a long region of repetitive DNA (termed the 70-bp repeats based on their approximate length) led to the predictions that these repeats are possible sites of the DNA lesions that initiate switching, or the source of DNA homology for *VSG* donor selection in recombination-based switching [17–19]. Often described as imperfect AT-rich 70-bp repeats, observations that this sequence also occurs proximal to *VSGs* within the genomic archive bolstered the *VSG* selection prediction [1]. Similarly, their predicted role in forming DNA lesions fell into disfavor when it was shown that gene conversion in trypanosomes grown *in vitro*, albeit at a very low frequency, does not require 70-bp repeats [20], favoring the proposed role in providing homology for recombination.

Yet, the proposed function of the 70-bp repeats was never experimentally tested. This was due, in part, to the inability to analyze these events due to low levels of switching that occur under laboratory conditions. Here, we artificially increase the rate of *VSG* switching to determine how the 70-bp repeats affect *VSG* donor selection during gene conversion. The data presented herein confirm that the 70-bp repeats can function to promote selection of *VSGs* from throughout the silent repertoire. In addition, an expanded analysis of the 70-bp repeat sequence enabled us to identify a minimal 70-bp repeat region that promotes archival *VSG* selection. In the course of this analysis we also discovered that the 70-bp repeats could have previously unreported affects on the frequency of *VSG* switching and cell cycle progression. Furthermore, our data showed that the 70-bp repeats can direct *VSG* selection away from other BESs, their closest homologs, and toward the genomic archive, which has mechanistic and physiological implications. Our findings suggest that the 70-bp repeat regions are required for the normal outcomes of *VSG* switching, and thus the ability of *T*. *brucei* to survive in its host during a chronic infection.

## Results

### Conservation of the 70-bp repeat sequence within and among the genomes of African trypanosomes

**Fig 1:**
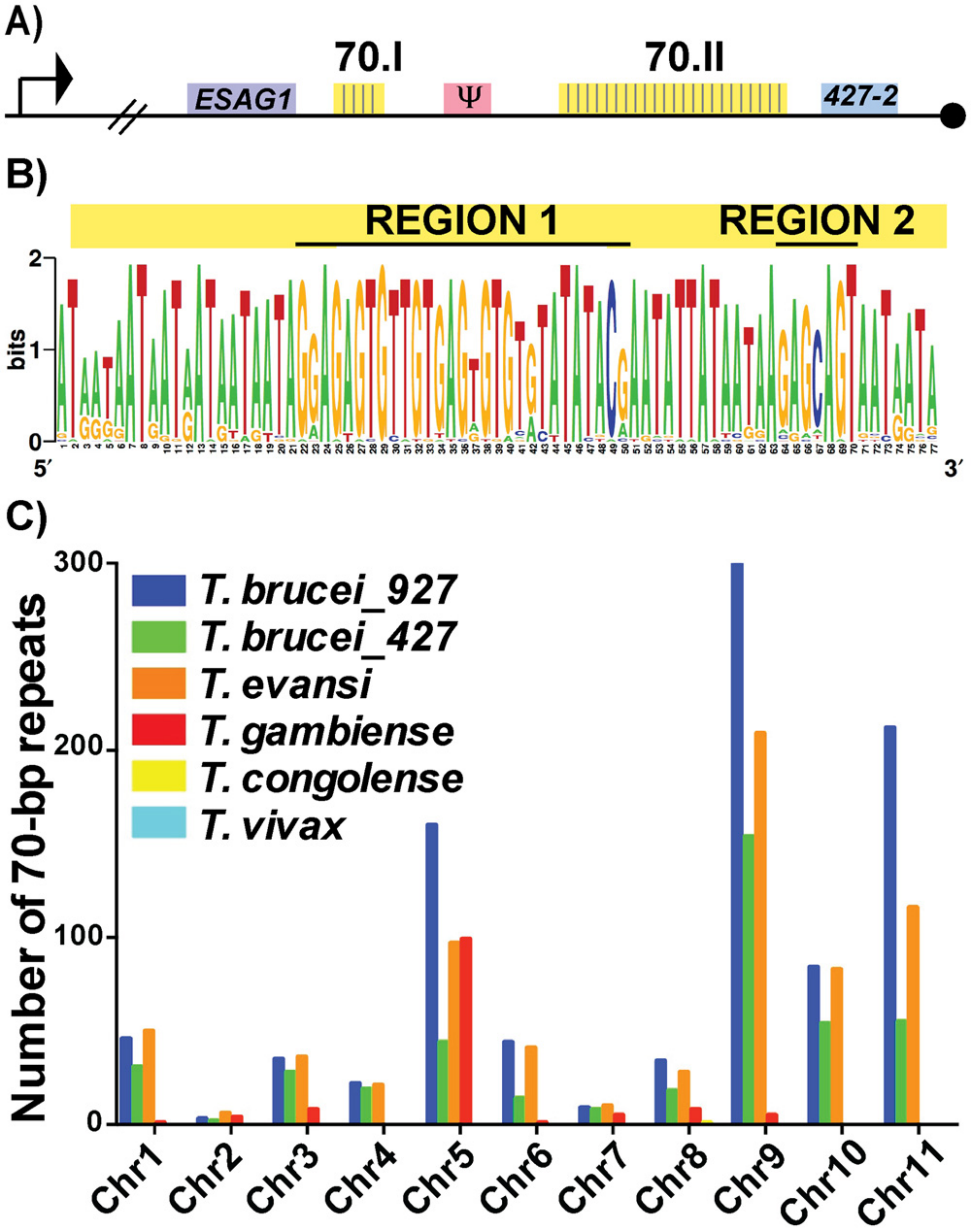
Conservation and genomic distribution of the 70-bp repeat sequence. A) Map of *T*. *brucei Lister 427* BES1 with promoter (bent arrow), terminal ESAG (ESAG1), *VSG* (blue), *VSG* pseudogene (pink), telomere (black circle), and 70-bp repeat regions (yellow) illustrated (70.I contains 3 repeats and 70.II contains 39 repeats). B) The identified 70-bp consensus sequence from shown as a logo created from the 42 repeats found in BES1 (weblogo.berkely.edu). C) Graph of the number of 70b-bp repeats (determined by e-value>40, identities> 70%, and length>40bp) enumerated per chromosome from the genomes of *T*. *brucei brucei TREU927* (blue), *T*. *brucei brucei Lister427* (green), *T*. *evansi STIB805* (orange), *T*. *brucei gambiense DAL972* (red), *T*. *congolense IL3000* (yellow), and *T*. *vivax Y486* (light blue).

To investigate the putative functions of the 70-bp repeats we first subjected the two repeat regions of *Lister427* BES1 (Fig 1A—70.I & 70.II) to fine mapping and the 42 identified repeat sequences were used to produce a consensus sequence logo (Fig 1B). Similar to previous studies of more limited sample sizes, the repeats were an average of 76-bp (usually running either 77-bp or 75-bp in length) and were AT-rich (78%). For the sake of consistency within the literature, the 70-bp repeat nomenclature will be maintained [17–19]. These data support previous work suggesting that the 70-bp sequence is highly conserved [18] and identified two pronounced GC-rich regions (Region1 and Region 2). Expanding the analysis to include repeat regions of additional BESs, within both *Lister427* [7] and *TREU927* (http://www.sanger.ac.uk/resources/downloads/protozoa/trypanosoma-brucei.html) genomes, showed that this conservation is consistent among *T*. *brucei* BES regions (S1 Fig and S1 Dataset). Thus, in the majority of BESs, a long region of conserved 70-bp sequence is maintained in close proximity to the sub-telomeric *VSG* gene.

Aside from the BES sequences from these two genomes, direct comparison of the frequency and organization of the 70-bp repeat sequence within available African Trypanosome genomes is limited by the variable quality of each genomic assembly, especially near the subtelomeric regions (http://tritrypdb.org/tritrypdb/). Operating within these confines, we sought to determine the prevalence of the 70-bp regions by performing a BLAST analysis of the consensus sequence against each chromosome of the available genomes (Fig 1C). While the 70-bp repeat sequence was not found in the genomes of South American trypanosome species, which do not undergo antigenic variation, it was abundant within the genomes of *T*. *brucei TREU 927*, *T*. *brucei Lister 427*, *T*. *evansi*, and *T*. *brucei gambiense* (a human-infectious subspecies). The abundance of 70-bp repeats in *T*. *evansi* (an emerging pathogen among livestock in the Middle East and Asia) was anticipated as its genome has extensive similarity with that of *T*. *brucei* [21]. The observation that *T*. *b*. *gambiense* has fewer 70-bp repeats per chromosome than the other *T*. *brucei* subspecies is difficult to interpret as it could be an artifact resulting from the sequencing of its genome (the genome of another human-infectious form, *T*. *b*. *rhodesiense*, has not been sequenced). In contrast, the absence of the 70-bp repeats from *T*. *congolense* and *T*. *vivax* could reflect real biological differences in antigenic variation between these very distinct species [22].

In addition to BESs and megabase chromosomes, *VSG*-containing contigs from T. brucei *Lister 427* minichromosomes contained the 70-bp consensus sequence in the proximity of *VSGs* (usually approximately 1.5 kb upstream) (S1 Table) [2]. Thus, the conserved 70-bp repeat sequence identified here is widely distributed among the genomes of African trypanosomes with anticipated positioning in long tracts on BESs and shorter tracts on the megabase and minichromosome arms in the proximity of *VSG* genes. The genomic conservation and distribution of this sequence lends support to the hypothesis that the 70-bp repeats contribute to homologous pairing and *VSG* donor selection during GC [20].

### 70-bp repeats promote *VSG* selection from diverse genomic sites

**Fig 2:**
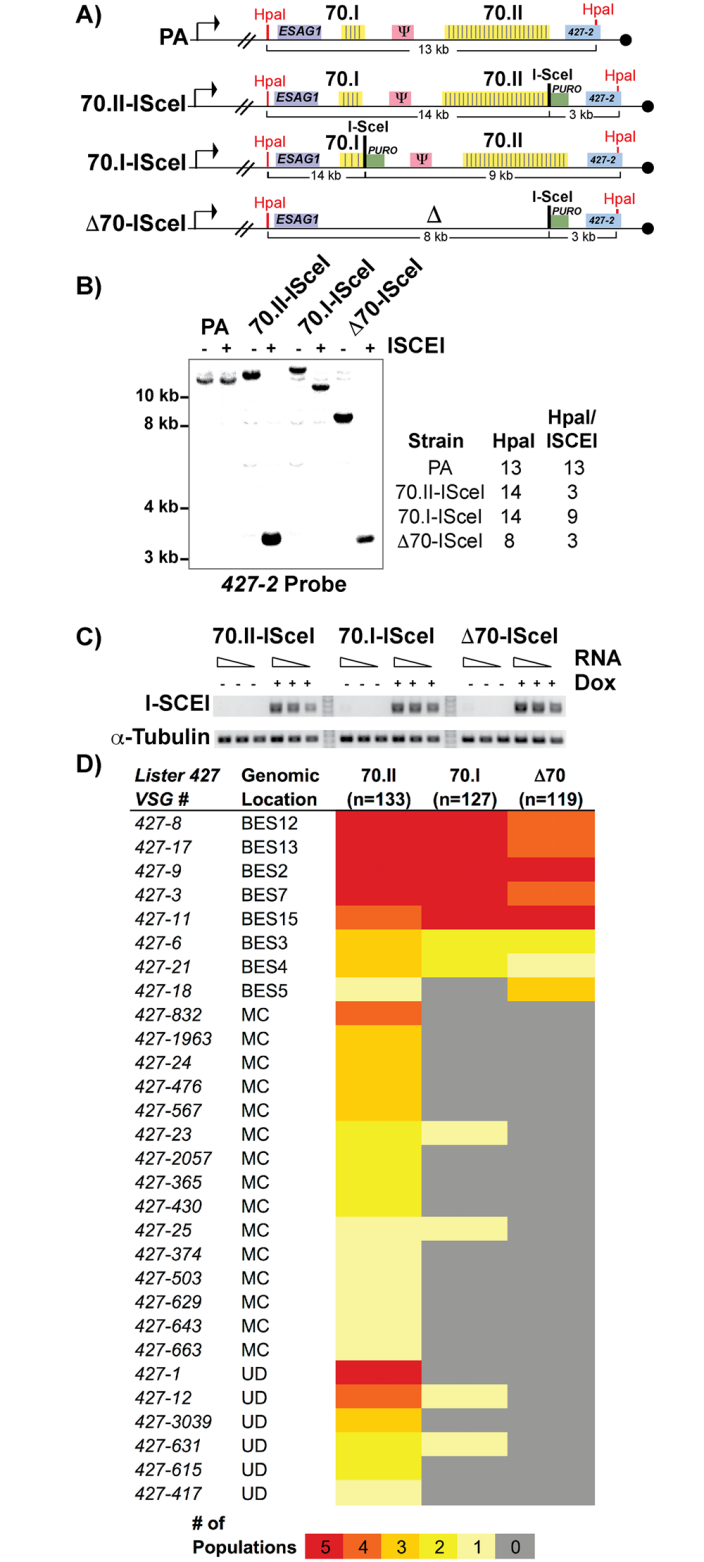
Effects of 70-bp repeats on *VSG* donor selection and switching. A) Map of BES1 modifications (in comparison to PA) used in analyzed strains illustrate ISceI site introduction with puromycin marker (green), HpaI restriction site location (red) used in Southern blot conformation is indicated along with anticipated sizes following digestion. B) Southern blots of representative clones from each BES1 modified line are shown for HpaI and HpaI/ISceI digestions probed with terminal BES1 *VSG* (427–2). C) Semi-quantitative RT-PCR of ISCEI enzyme expression for a 5-fold dilution of input RNA under non-inducting (- Dox) and inducing (+ Dox) conditions alongside a tubulin control. D) Heat map showing the number of populations in which clones (n = number of clones analyzed per strain) expressing a specific *VSG* arose: *Lister427 VSG reference number* and the predicted genomic locus (BES, MC, or undetermined [UD]) of the *VSG* are shown together with the number of populations (out of 5 total), in which it arose as depicted by color intensity using the color key (bottom).

To test this hypothesis, we sought to genetically manipulate the 70-bp repeats of the active BES and monitor the effects on switching, but were hindered by the naturally low frequency of *in vitro* switching (~1x10^-6^) in the *Lister 427* strain. We therefore established cell lines in which DNA double-stranded breaks (DSB) could be induced in the actively expressed BES, to increase the depth of analysis by increasing the frequency of switching by GC [12,14]. An ISceI enzymatic cleavage site was introduced into BES1 proximal to a long region of repeats (“70.II-ISceI”, 39 repeat iterations), a short region of repeats (“70.I-ISceI”, 3 repeats) and in a repeat deletion mutant (“Δ70-ISceI”, no repeats) (Fig 2A; oligos used for constructs are in S1 Text). The veracity of the ISceI cleavage sites was confirmed by Southern blot analysis and the consistent expression of the ISCEI enzyme among lines confirmed (Fig 1B and 1C). Five populations of each ISceI-bearing strain (70.II-ISceI, 70.I-ISceI, or Δ70-ISceI) were grown for 3 days under normal (- doxycycline) or DSB-inducing (+ doxycycline) conditions, and cells that had switched from their initial *VSG* (*427–2*) to an alternative *VSG* gene were isolated over magnetic cell-sorting (MACS) columns, as described [12,13] (experimental pipeline details S2 Fig). The resulting *VSG*-switched cells were cloned by limiting dilution and the resulting clones were used to determine both the mechanism of switching (using established genetic methods [13,23]) and to identify the newly expressed *VSG* (using traditional RT-PCR followed by sequence analysis and VSGnome BLAST alignment at http://tryps.rockefeller.edu) for more than 100 clones from each line (S2–S4 Tables). As anticipated, based on previous studies [12,13], following DSB induction, all lines switched by GC and preferentially favored the selection of BES-encoded *VSG* donors (Fig 2D). Notably, when the 70-bp repeat region proximal to ISceI was long (70.II-ISceI), 48% of the selected *VSGs* arose from minichromosomal (MC) or undetermined sites (UD) as opposed to homologous BESs (Fig 2D). In contrast, ISceI break formation proximal to a very small repeat region (70.I-ISceI) or after repeat deletion (Δ70-ISceI) resulted in the selection of BES encoded *VSGs* in 98% or 100% of clones, respectively (Fig 2D). Thus, short or deleted 70-bp repeats appeared defective in selecting *VSGs* from the *VSG* genomic archive when compared with longer 70-bp repeat regions.

### Gene conversion replaces defective 70-bp repeat regions

**Fig 3:**
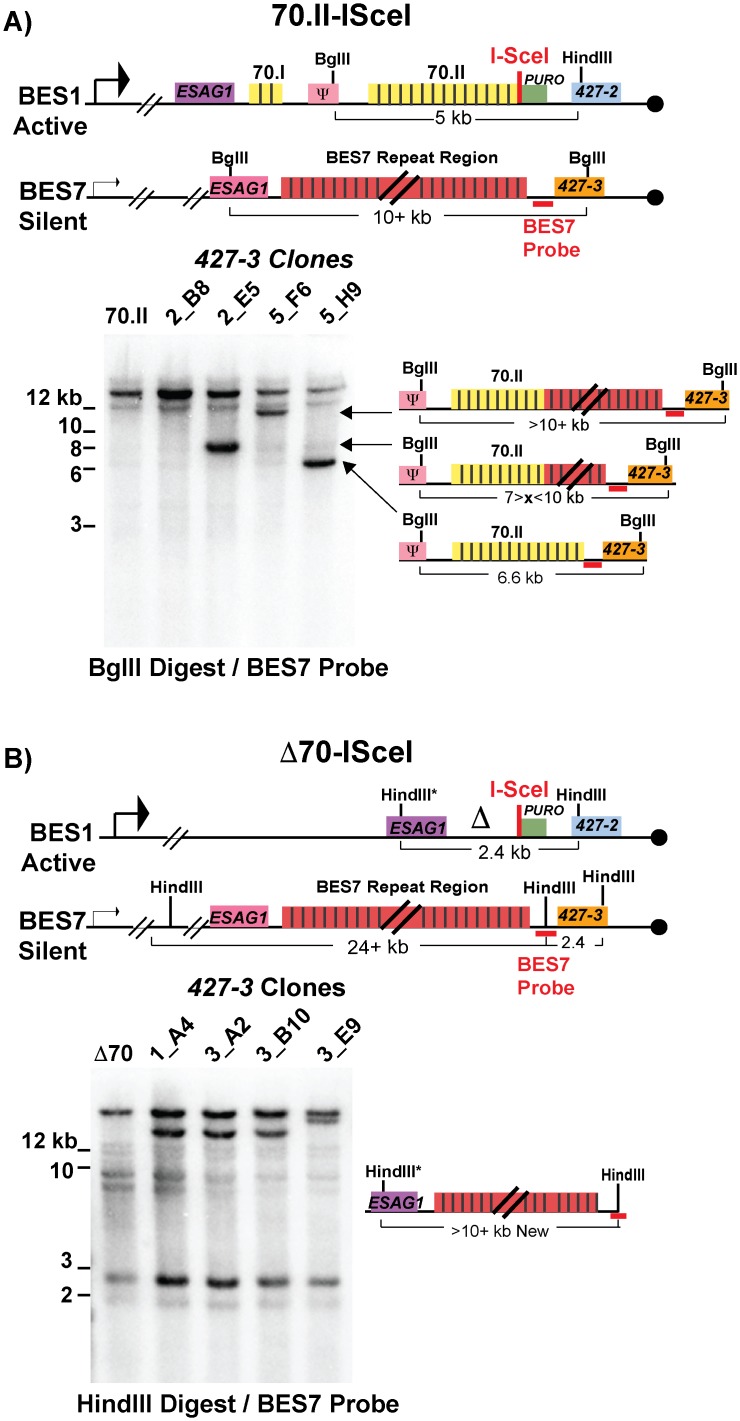
BES1 repeat content following GC with BES7. Field-inversion gel electrophoresis and Southern blot analysis of *VSG427-3* switched clones arising from A) BES1-70.II-ISceI or B) BES1-Δ70-ISceI GC with silent BES7 donor digested BglII digest or HindIII, respectively. BES7 probe (red bar) proximal to *427–3* results in the formation of a new band whose size approximates the region of BES7 transferred during gene conversion. Diagrams to the right of Southern blots show the predicted composition of the newly formed band.

Following a DSB, either naturally occurring or induced, single-stranded DNA is liberated initiating a homology search that is likely resolved by break-induced replication. Genetic analysis of individual switched clones can determine the extent of DNA transferred from the donor site into the active BES during GC. One of the most common switching events observed was between BES1 and BES7, resulting in the expression of *VSG427-3*. Using a BES7 probe upstream of the *VSG*, clones that have recombined *VSG427-3* into BES1 will form a new band (upon appropriate restriction digestion) whose length indicates the region of BES7 transferred during GC. The resulting data indicate how GC affects 70-bp repeat maintenance or the recovery of defective repeat regions (Fig 3).

Clones arising from a BES1 with normal 70-bp repeats (Fig 3A—70.II-ISceI) showed a variety of outcomes that included the addition of no new repeats (5_H9 = 6.6 kb), partial addition of BES7 repeats (2_E5 ~8 kb & 5_F6 > 10 kb), or the translocation of full length BES7 repeats (2_B8 > 12 kb). In contrast, when BES1 harbors no 70-bp repeats (Fig 3B—Δ70-ISceI) the full region of BES7 repeats was consistently incorporated into BES1 during switching (1_A4, 3_A2, & 3_B10 > 12 kb). In one clone it appears that a region larger than the BES7 repeats was incorporated into BES1 (3_E9); similar long-range recombination events have been reported during GC switching in other studies [23]. It should be noted that the determination of the precise lengths of the regions transferred from BES7 to BES2 is hindered by the fact that the exact length of the repeats encoded in BES7 is unknown. Thus, we observe that the 70-bp repeat region in the active BES can be repopulated, maintained, or extended during GC-based recombination with another BES.

### Effects of 70-bp repeats on growth, cell cycle progression, and *VSG* switching

**Fig 4:**
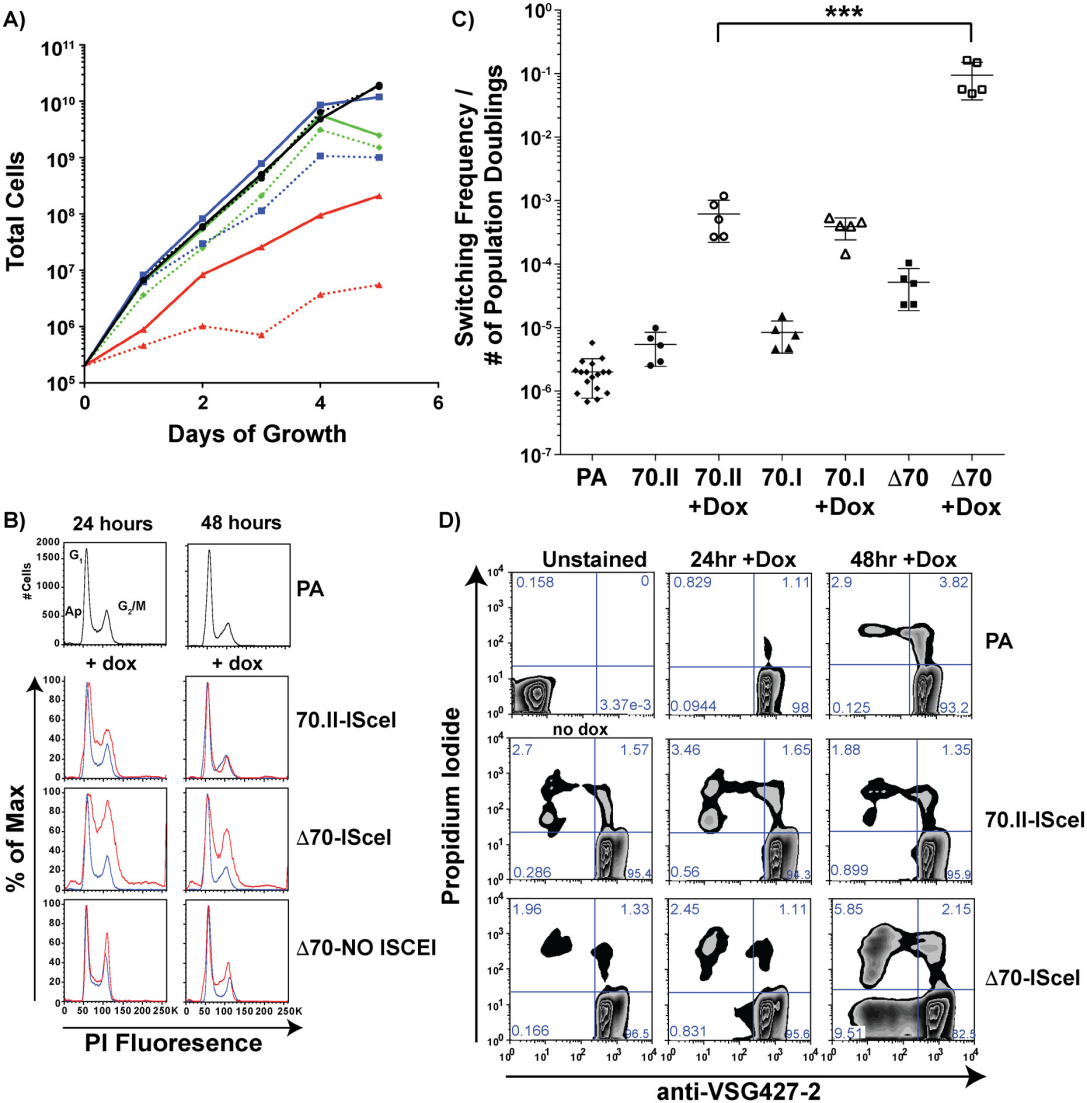
Growth and *VSG* switching in 70-bp repeat analysis lines. A) Growth of 70-bp repeat-analysis strains is shown over 5 days of consistent cell passage with (dashed lines) and without (solid lines) doxycycline induction for the ISCEI-bearing parental line (black ●), 70.II-ISceI (blue ■), and Δ70-ISceI (red ▲), as well as Δ70-No ISceI (green ◆). B) DNA content frequency histogram measured by PI fluorescence used to estimate the percentage of cells in G_1_ and G_2_/M at 24 & 48 hours following doxycycline induction for ISceI bearing strains (red) in comparison with parental strain (blue). C) Switching frequencies of 70-bp repeat analysis strains (PA = ♦, 70.II = ●, 70.I = ▲, Δ70 = ■) during growth with (filled symbol) or without (hollow symbol) doxycycline induction (three days) is normalized to the number of population doublings (***<0.0001, F-test) as determined from growth data in panel A. D) Flow-cytometry analysis of proportion of cells without *VSG427-2* (x-axis) and staining with propidium iodide (y-axis), as a measure of *VSG* switching and cell death, respectively, shown as a zebra plot with frequencies of each population per quadrant shown.

The growth rate and frequency of *VSG* switching following DSB induction could affect the number of *VSG* donors selected. To verify that the *VSG* donor selection phenotypes reported in Fig 2 were dependent solely on the effect of the 70-bp repeat regions, cellular growth and *VSG* switching were monitored in these lines. Following doxycycline induction, all lines harboring an ISceI site in BES1 displayed a growth defect when compared to the parental line. For strains with intact 70-bp repeats (Fig 4A—70.II-ISceI [blue lines]) the delay in growth was modest, yet DSB formation in the 70-bp deletion mutant (Δ70-ISceI) exacerbated a pronounced preexisting growth defect (Fig 4A—Red lines). We predicted that the growth defect observed without doxycycline induction resulted from leaky expression of the ISCEI enzyme (a known complication of expression from the rDNA spacer [24]), and tested this prediction using a 70-bp repeat deletion mutant that did not harbor the ISCEI enzyme (Δ70-NO ISCEI). Deletion of the repeats from BES1 did not result in a growth defect in the absence of the ISCEI (as anticipated from previous work on a similar construction [20,25]). Thus, the observed growth defects in ISCEI-expressing lines appear to result from DSB formation in the active BES, which was most pronounced when the 70-bp repeats were deleted.

Because DSB formation can activate a cell cycle checkpoint (reviewed in [26,27]) and the Δ70-ISceI cell line has a growth defect, the effects of DSB formation on the cell cycle were examined in these lines. Cells harboring a DSB site near wild-type 70-bp repeat regions resulted in a minor cell cycle delay at 24 hours that was largely resolved by 48 hours (Fig 4B—70-II-ISceI). In contrast, deletion of the BES1 70-bp repeats resulted in a severe cell cycle defect that was only partially resolved at 48 hours post-induction (Fig 4B—Δ70-ISceI). To determine if the defect results from DSB formation, cell-cycle progression was monitored in the 70-bp repeat deletion mutant that does not harbor ISCEI (Δ70-No ISCEI). These cells did not have the cell cycle defect observed in ISCEI-expressing lines at 24 hours, but did display minor accumulation of cells in S-phase at 48 hours post-induction (this could result from naturally occurring breaks arising late in growth that are not resolved normally in this line). Together these data indicate that the growth delays observed in these cell lines are associated with cell cycle defects arising from DSB formation and suggest that the deletion of 70-bp repeats from the active site exacerbates these defects.

Multiple studies have shown that induction of ISceI-induced breaks in the active BES results in increased *VSG* switching, but the precise amount of switching can vary depending on the location of DSB formation an the activity of the ISCEI enzyme [12,14]. To determine if the diversity of *VSG* gene selection (reported in Fig 2) resulted from differences in switching dynamics, the *VSG* switching frequency was quantified for the ISceI-bearing cell lines and normalized to the number of population doublings (Fig 4C, normalization derived from Fig 4A). DNA break formation proximal to the long repeat region (Fig 4C—70.II) resulted in approximately 100-fold increase in switching, compared to wild-type cells, as previously observed [12]. DSB formation in the proximity of only three 70-bp repeats (70.I) resulted in a similar switching frequency, but a vastly different diversity in the selected *VSGs* (98% BES encoded *VSGs* selected compared with 52% in the 70.II cell line [Fig 2]). This comparison underscores the role of the 70-bp repeat region in selection of *VSGs* from the genomic archive. However, deletion of the 70-bp repeats resulted in a switching frequency, upon DSB induction, that was 10–100 fold greater than strains harboring 70-bp repeats (Fig 4C—Δ70-ISceI), such that 1 in 10 cells had switched (a frequency observable by flow-cytometry alone [Fig 4D]). This was in contrast with the previous report of a similarly constructed strain [12], probably because the slow growth phenotype had, in the previous study, led to the selection of a clone in which the ISceI site or enzymatic function was lost. The switching frequency calculated after DSB formation in the 70-ISceI line is likely affected by the observed growth and cell cycle defects, so is not directly comparable to the values calculated for isogenic lines containing repeats, where DSB-induction does not noticeably affect growth and cell-cycle progression. Nonetheless, the diminished capacity for *VSG* donor selection in the Δ70-ISceI line was definitely not the result of a reduction in switching frequency.

### Identification of a minimal functional 70-bp repeat sequence

**Fig 5:**
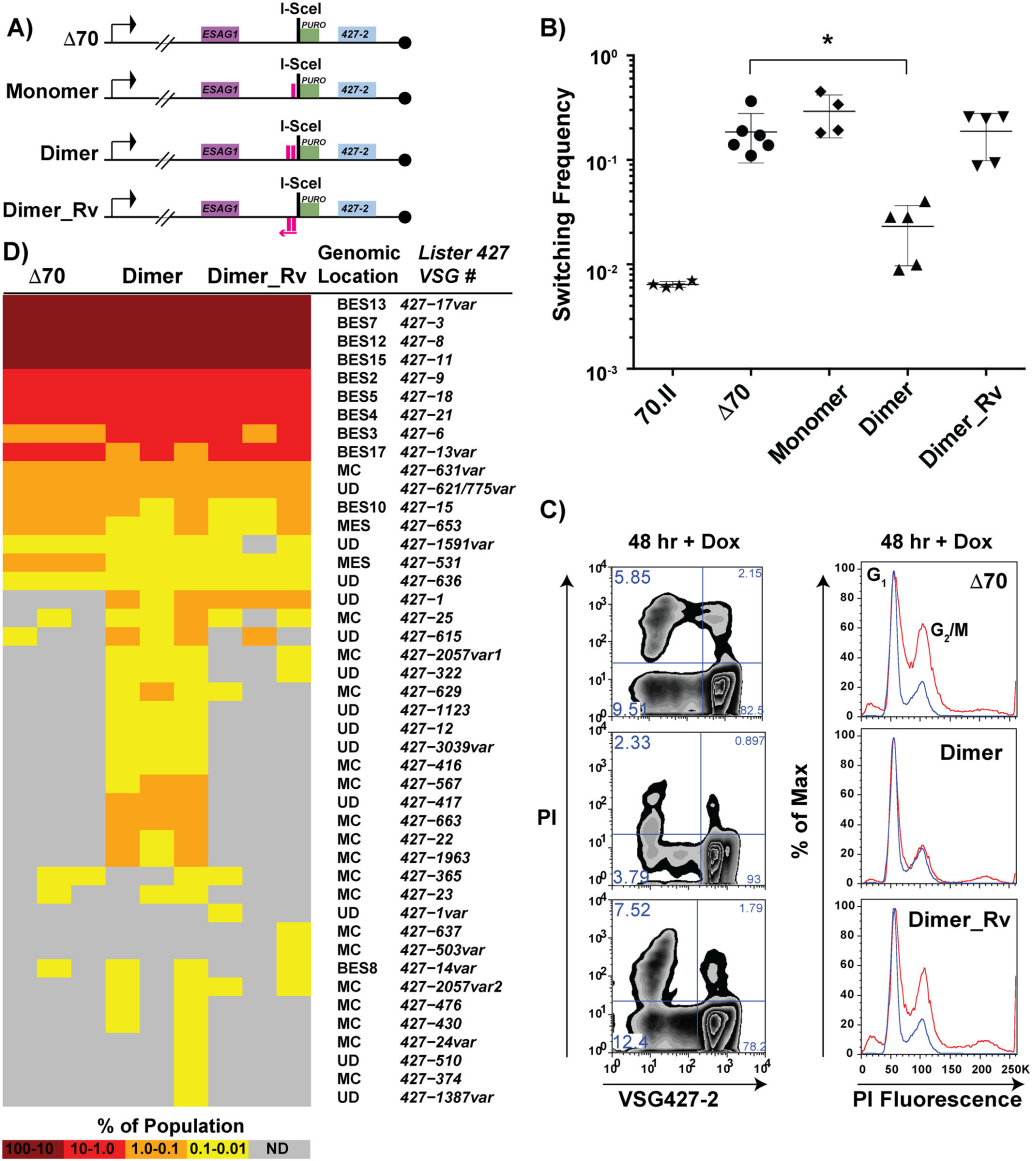
Effects of engineered 70-bp repeat sequences on *VSG* switching and donor selection. A) BES1 maps of cell lines bearing repeat deletions (Δ70) and introductions, shown as pink boxes, or in reverse (pink arrow). B) Observed switching frequencies of doxycycline induced strains bearing alternative repeat regions: wild-type repeats (70.II, ★), no repeats (Δ70, ●), monomeric motif (Monomer, ◆), dimeric motif (Dimer, ▴), and reverse dimeric motif (Dimer_Rv). Difference in switching between Δ70 and Dimer motif introduction is statistically significant (asterisk over bracket pval = 0.027). C) Flow-cytometry analysis of switched cells (427–2 negative population) and cell cycle (DNA content frequency histogram measured by PI fluorescence) at 48 hours following doxycycline induction. D) VSG-seq analysis of MACS-isolated switchers from three biological replicates of Δ70, Dimer, & Dimer_Rv is shown in the form of a heat diagram where the color intensity reflects the proportion of each *VSG* RNA in the population. The *Lister427* VSG number and its predicted genomic location are shown on the right of the heat diagram where var indicates that the assembled *VSG* sequence had minor sequence variations from the most similar 427 reference *VSG*.

Based on the observation that the 70.I-ISceI has a normal switching frequency and a modest capacity for archival *VSG* donor selection, we predicted that a minimal 70-bp repeat region could recapitulate the phenotypes associated with the long, cognate 70-bp repeat regions. To test this prediction, the conserved 70-bp repeat sequence presented in Fig 1 was used to design synthetic 70-bp regions, which were introduced into the Δ70-ISceI landscape to produce stable cell lines and analyze their phenotypes. The resulting cell lines, which harbor discrete repeat regions proximal to the ISceI site, are as follows: “Monomer”, which bears a single 70-bp repeat; “Dimer”, consisting of two monomeric units separated by a cognate spacer (ATAATA); and “Dimer_Rv”, which harbors the Dimer sequence in the opposite orientation with respect to transcription (Fig 5A, repeat insertion sequences shown in S1 Text). DSB induction in the Dimer cell line reduced the *VSG* switching frequency nearly 10-fold from Δ70-ISceI levels (2.3x10^-2^ compared with 1.9x10^-1^, respectively), where strains harboring the 70-bp repeat Monomer or Dimer_Rv sequences were unchanged from the deletion mutant (Fig 5B). Similarly, the growth and cell cycle defects observed in the absence of 70-bp repeats (Fig 4—Δ 70-ISceI) were significantly improved by the addition of the Dimer region, while this was not the case for Monomer or Dimer_Rv lines (Fig 5C and S3 Fig). (Phenotypes of an additional mutated repeat line “Mut_Dimer” did not suppress the Δ70-ISceI phenotypes [shown only in S3 Fig]). If *VSG* switching and cell growth phenotypes correlate with *VSG* donor selection (as suggested by data in Figs 2 and 3), we would expect the Dimer cell line to result in selection of *VSGs* from within the genomic archive.

To determine the effect of the synthetic repeat sequences on *VSG* donor selection at an increased depth, RNA was extracted from DSB-induced post-MACS eluates from biological triplicates of these lines for VSG-seq analysis [28]. The VSG-Seq method is distinct from the clonal analysis of *VSG* donor selection presented in Fig 2 in that it permits the identification of *VSG* RNAs comprising as little as 0.01% of the population [28]. At this sensitivity, we observed that the line lacking repeats in the active BES (Δ70-ISceI) could occasionally select *VSGs* from sites other than BESs (Fig 5D, supported by data in S5 Table), including two from metacyclic expression sites (MES), one from a MC, and four from other undetermined (UD) loci. Introduction of the repeat Dimer resulted in a significant (pval = 0.0026) increase in the number of *VSGs* selected when compared with the no-repeat line (average of 18 *VSGs* in Δ70-ISceI and 35 *VSGs* in Dimer populations, SI 9). This near doubling in the diversity of *VSG* selection was the result of a substantial increase in MC *VSG* selection (pval = 0.005, average Δ70-ISceI = 1 MC & Dimer = 11 MC) and a more modest, but statistically significant, increase in the selection of *VSGs* arising from undetermined loci (pval = 0.001, average Δ70-ISceI = 4 UD & Dimer = 12 UD). In contrast, addition of the Dimer_Rv sequence did not result in a significant increase in the selected *VSG* repertoire (pval = 0.205), although some subtle differences between Δ70-ISceI and Dimer_Rv strains can be observed (Fig 5D). These data have identified a minimal 70-bp repeat region able to partially suppress the collection phenotypes (i.e. cell growth defect, cell cycle delay, increased *VSG* switching, and reduced *VSG* donor selection) associated with DSB formation proximal to a 70-bp repeat deletion mutant and result in phenotypes similar to lines harboring cognate 70-bp repeats.

## Discussion

While unbalanced chromosomal translocations can fuel evolutionary change, they are generally deleterious to eukaryotic, especially mammalian, genomes. African trypanosomes are a useful model of chromosomal translocations because their essential pathogenic process, antigenic variation, depends on them. The early observation that genetic transposition of a new *VSG* into the active BES terminates within a tract of repetitive DNA inspired passionate functional speculation. Yet, the available sequence information and genetic tools of the time (and of studies that followed in the 1990s) restricted the scope of possible analyses. Thus, a viable hypothesis, that the repeats provide homology for recombination, became widely accepted [29,30] but was not tested. In the present study we applied a variety of recently available sequencing databases (BESs, trypanosome genomes, and VSGnome), a next-generation sequencing method (VSG-seq), genetic tools (including ISceI DSB induction), and cell biology assays (such as *VSG* switching frequency quantification) to test this long-standing hypothesis.

Classic sequencing approaches of the mid-1980s allowed three groups to determine the essential characteristics of the 70-bp repeats [17–19] and analysis of cosmid clones suggested that *VSG* genes and 70-bp repeats were widely distributed in the genome [31]. Completion of the first African trypanosome genome sequencing project (*TREU927*) confirmed, in detail, that the 70-bp repeat sequence is not only found at the BES subtelomeres but also proximal to *VSGs* on the chromosome arms [1]. Yet, at that time, determining the degree of 70-bp repeat conservation within the genome was hindered by inherent challenges associated with assembling the sequences at the ends of chromosomes. Here, we utilized existing comprehensive BES sequence data (ABI 3730, with approximately 700-bp read length [7]) to produce a 70-bp consensus sequence and confirm its degree conservation among numerous BESs. The length and conservation of this sequence corroborates some early findings [18], but disagree somewhat with the often-asserted position that the 70-bp repeats are imperfect and have variable length [7,30,32,33]. While the length of the AT-rich regions between conserved repeating units can vary, as reported [19], we would suggest that the data presented in this study highlight the significance of the conserved repeating unit presented in Fig 1. It is important to note that the findings reported here do not address the putative function of the repetitive regions in DNA instability, the proposed function of the triplet repeats [19,34].

The order and conservation of the 70-bp repeats inspired us to revisit the question of function. Previous deletion of the BES1 70-bp repeat regions showed that the repeats themselves are not required for the low levels of gene conversion observed *in vitro* [20]. This finding was significant in that it challenged long-held speculation that the repeats function as specific endonuclease-cleavage sites. The recent availability of the *Liste r427* VSGnome (sequences of all *VSG* genes within the genomic archive) [2] enabled testing of the second predicted function of the 70-bp repeats, namely providing homology in *VSG* donor selection. However, the amount of switching that occurs *in vitro* is too low (1x10^-6^) to permit a substantive analysis of *VSG* switching outcomes. This limitation was overcome through utilization of an artificial DNA breaking system that has been shown to increase the *VSG* switching frequency [12,14], which occurs by gene conversion, in a similar manner to those that occur through more natural DNA break systems analyzed [13]. Use of the established ISceI endonuclease cleavage system for DSB formation enabled in-depth analysis of how different regions, and mutations, of 70-bp repeats affect *VSG* switching and its outcomes. The caveat, of course, is that ISceI is an artificial system and limits our interpretation of the implications for naturally occurring infections. Nonetheless, this genetic tool enabled the observation of genetic phenomena that would not have been detectable otherwise. Thus, individual clonal analysis of switched cells using the VSGnome resource allowed us to demonstrate that the BES encoded repetitive regions are required for selection of a normal repertoire of *VSG* genes, the first observed phenotype for the 70-bp repeats.

The increase in switching frequency following DNA break formation in the BES1 constructions presented here also enabled us to observe unexpected outcomes of 70-bp repeat deletion and variations. Among these cell lines we observed that the 70-bp repeats have previously unappreciated and apparently connected effects on cell growth, cell cycle progression, and *VSG* switching following DSB formation. The observation that deletion of 70-bp repeats results in significant cell cycle delays following DSB formation could suggest that, in comparison to lines harboring wild-type repeats, this cell line is defective for DNA break repair. The fact that the same mutant cell line also switches much more frequently and results in increased cell death may suggest that cell lines harboring functional repeats process the DNA breaks more efficiently, as evidenced by the minimal cell cycle delay at 24 hours in 70.II-ISceI. These effects appear to depend on ISceI-induced DSB formation, as shown by the Δ70-No ISCEI cell line, whose behavior was largely unaffected by the repeat deletion, as expected from the literature [20]. This collection of phenotypes was consistent among all cell lines that harbor “functional” (70.II-ISceI, 70.I-ISceI, and Dimer) vs. “dysfunctional” (Δ70-ISceI, Monomer, & Dimer_Rv) 70-bp repeat regions. Alternatively, similar phenotypes might be observed if the 70-bp repeats affect the ISceI cutting efficiency. This could occur if there was steric hindrance at the cut site, which could result from binding proteins or DNA secondary structure. Further exploration of the phenotypic alterations associated with the 70-bp repeat variations could lead to new mechanistic understanding of the requirements for the chromosomal translocations that support *T*. *brucei* antigenic variation.

Diverse pathogens utilize antigenic variation to escape the host immune system; among them *T*. *brucei* has the most extensive archive of surface antigen genes (the *VSGs*) [35]. Yet, the extensive repertoire of *VSG* genes would be useless if they could not be activated. Here we have shown that the 70-bp repeats are a key feature that permits access to the *VSG* archive. This result experimentally validates previous speculations and extends our understanding by highlighting the specific DNA element, sequence, and orientation required for selection, at a depth of analysis only recently made possible by VSG-seq [28]. It is unclear at this time if the 70-bp repeats influence the formation of new *VSG* gene variants through mosaicism, a known from of repertoire expansion mediated by recombination within *VSG* coding sequences. At the sensitivity of VSG-seq and the discriminatory ability of its cognate assembly component, genes identified as VSG-variants (Fig 5D—“var”) appear to share similarities with mosaic *VSGs*.

BESs are essentially long homologous regions that not only contain the same organization, genes, and genetic elements, but are also nearly identical to one another at the sequence level for more than 50 kilobases [7]. As homology length generally determines the frequency of recombination [36], it is not unexpected that homologous BESs (harboring many kb of 70-bp repeats) are primary genomic sites favored during *VSG* selection. What is surprising is the extent to which regions of wild-type repeats select sites other than BESs (48% of *VSGs* selected following induction of 70.II-ISceI). While *VSG* donor selection could be based on homology alone, our findings raise the possibility that another external factor, acting on the 70-bp repeats, promotes selection of non-BES encoded *VSGs*. This effect could be in the form of repeat-specific DNA-binding proteins or be associated with subnuclear positioning during gene conversion, which is known to affect *VSG* expression [37]. Overall, this study demonstrates that, following DSB formation and subsequent liberation of ssDNA, the 70-bp repeats guide homologous pairing toward diverse genomic sites that harbor the *VSG* archive, a repertoire-expansion function that is crucial to the long-term survival of the parasite in its host. While the intricacies of *VSG* switching may be unique to African trypanosome parasites, the genetic processes described here have implications for chromosomal translocations that occur within other eukaryotic genomes.

## Methods

### Analysis of repeat sequence conservation and genomic distribution

The conserved repeat sequence was identified visually based on the BES sequences from *T*. *brucei Lister 427*, and logos produced by http://weblogo.berkeley.edu/logo.cgi. The same approach was then applied to *TREU927* BES sequences (S1 Fig and S1 Dataset). The consensus sequence from the BES1 repeat logo was used to BLAST (http://blast.ncbi.nlm.nih.gov/Blast.cgi) the *TREU927* genome and hits were called based on Max Score and Percent Identity. *VSG* proximity was called based on the *TREU927* genome annotation. Contigs resulting from deep sequencing of the MC DNA fraction were used to determine repeat conservation and distance with respect to MC *VSG* genes [2]. The sequence of each of the 11 megabase chromosomes from *TREU927 (T*. *brucei brucei)*, *Lister427 (T*. *brucei brucei)*, *DAL972 (T*. *brucei gambiense)*, *IL3000 (T*. *congolense)*, *STIB805 (T*. *evansi)*, *and Y486 (T*. *vivax)* genomes were downloaded from http://tritrypdb.org/tritrypdb/ and BLASTed (http://blast.ncbi.nlm.nih.gov/Blast.cgi) against the 77 bp consensus sequence (Fig 1A). Hits were counted as 70-bp repeats if their length was greater than 45 bp, had and e-value was greater than 40, and their identity was greater than 70%.

### *Trypanosoma brucei* line constructions, growth, and cell cycle analysis

Cell lines were generated from *Lister427* bloodstream-form trypanosomes derived from the “single marker” (SM) line [38]. “Wild-type” in this study was SM with a blasticidin-resistance gene inserted at the active BES1 promoter, which can be put under blasticidin selection to prevent BES transcriptional switching, as was done in the present study only to stabilize the population until the time of DSB induction. The parental line (PA) of all ISCEI introduction experiments is “SM-NLS-ISCEI-HA” [12], which has a copy of a tetracycline-inducible ISCEI enzyme encoded in the rDNA spacer region and a hygromycin resistance marker incorporated at the BES1 promoter. The ISceI cut site and recombinatorial homology to specific locations of BES1 were added to a puromycin selection cassette by PCR (oligos found in S1 Text), cloned into a pGEMT vector, and DNA fragments were liberated by digest prior to transfection using the AMAXA Nucleofector [39]. Sequences were confirmed and DNA fragments librated from the vector for AMAXA transfections. Transformants were selected in 10 μg/mL puromycin, screened for BES1 incorporation by PCR, and confirmed by Southern blot analysis. Semi-quantitative RT-PCR was performed using Superscript III for cDNA amplification as described (thermofisher.com) followed by 25 cycles of PCR amplification using taq polymerase. Cell lines were cultured *in vitro* in HMI-9 medium at 37°C [40] and ISCEI induced using 1μg/mL doxycycline (dox). Strain growth was monitored by continuous passage by diluting daily to 1x10^5^ cells and measuring additive growth over 5 days. Standard flow-cytometry approaches were used to measure cell death and cell cycle progression using propidium iodide [41].

### Southern blot analysis

DNA restriction fragments were separated by either standard agarose gel electrophoresis (1–12 kb) or Field Inversion Gel Electrophoresis (FIGE) (1–25 kb) using established methods. Southern blots were produced using capillary blotting and neutral transfer paper (GE Scientific). DNA probes were made by PCR amplification, ^32^P-radiolabeled using Prime-It II Random Labeing Kit (Stratagene), and purified over G-50 microcolumns. Blots were probe-hybridized, washed and visualized by phosphorimaging (GE Healthcare).

### Isolation and analysis of switched clones

An experimental pipeline was established for the direct comparison of switching frequency, mechanism, and *VSG* donor selection (S2 Fig). Cells were grown from 5,000 cells to 50 million cells in media with or without doxycycline. Approximately 50 million cells were harvested and depleted over magnetic-activated cell sorting columns (MACS) using anti-Lister427 VSG-2 antibody (monoclonal antibody available for order through Memorial Sloan Kettering Cancer Center https://www.mskcc.org/research-advantage/core-facilities/monoclonal-antibody-core-facility) as described previously [13]. Half of the resulting “switcher-enriched” cells were used to quantify switching by flow-cytometry (measuring the number of switched cells as a proportion of the total population, as previously described [12]) and the other half was plated to limiting dilution and single cell clones were recovered and replica-plated for genetic analysis (similar to previous studies [13,23]), RNA extraction and *VSG* analysis, or long-term storage. Mechanisms of switching were determined by a combination of genetic tests and antibiotic sensitivity, as described [13,23]. RNA from clones was used to make cDNA, and the *VSG* was amplified by RT-PCR and sequenced directly from PCR products. The resulting sequence for each clone was aligned to the VSGnome database BLAST server (http://129.85.245.250/index.html) to identify the top *VSG* hit [2].

### VSG-seq analysis of 70-bp repeat modified strains

Cell lines bearing BES1 70-bp deletion or alterations were doxycycline induced for ISCEI DSB formation and the resulting switched cells isolated by MACS, as described above. RNA was extracted from three biological replicate populations of each induced strain and this material was used to prepare VSG-seq libraries [28].

A reference *VSG* database was created from *VSG* sequences assembled with the *de novo* assembler Trinity [42]. Trinity was first run on each library individually, and then run on libraries grouped by condition. All open reading frames (ORFs) were identified in each assembled contig, where an ORF is defined as a start codon to stop codon, a start codon to the end of a contig, or the beginning of a contig to a stop codon. BLASTn (v2.2.28+) was used to identify *VSG* ORFs [43] and ORFS with an alignment to a 427 *VSG* sequence with an e-value of < 1e-10 were considered true *VSG* sequences. The sets of *VSG* sequences from all assemblies were then merged using cd-hit-est (cd-hit v4.6.1) [44,45], with the parameters *-c 0*.*98 -n 8 -r 1 -G 1 -g 1 -b 20 -s 0*.*0 -aL 0*.*0 -aS 0*.*5*. Final assembled VSG sequences were all checked against NCBI’s nr/nt database using BLASTn.

Once reference sequences were determined, quantification was performed as described previously [28]. Noise (*VSGs* measured below the limit of detection, 0.01%) and contamination (the starting *VSG*, *427–2*) were removed. The relative abundance of each remaining expressed *VSG* was then calculated using its measured FPKM (fragments per kilobase of transcript per million mapped reads). To evaluate donor selection with respect to genomic position, each of these expressed VSGs was then compared to the Lister427 VSGnome database (http://129.85.245.250/index.html). Assembled *VSG* sequences, when compared to the most similar 427 *VSG*, were identified as the 427 *VSG* when they had either 100% identity over >99% of the length of the assembled ORF or >99% identity over 100% of the assembled ORF. Otherwise, assembled *VSGs* were referred to as variants (“var” in Fig 5) of the most similar Lister427 *VSG*. These data were then used to create a heatmap using heatmap.2 from the gplots package in R (https://cran.r-project.org/web/packages/gplots/gplots.pdf).). The VSG-seq data have been deposited in the SRA database under the project number SRP062141.

## Supporting Information

S1 Fig 70-bp repeat logos. The consensus DNA repeat is shown as a logo [weblogo.berkely.edu] produced from the repeat motifs in three BESs from *Lister427* and two BESs from *TREU927*.
(JPG)

S2 Fig Pipeline for isolation and analysis switched clones. Cultures grown for switching analysis are depleted of cells harboring the original VSG coat by MACS analysis were split determine switching frequency by flow-cytometry (as described previously) and for single cell cloning. Switched clones were validated by high-throughput FACS (HTS-FACS) analysis and their mechanisms of switching determined using a set of genetic criteria. RNA was extracted from each clone and subjected to One-step RT-PCR followed by direct PCR sequencing and VSGnome Blast of the PCR products.
(TIF)

S3 Fig Cell cycle and growth of repeat introduction strains. A) DNA content frequency histogram measured by PI fluorescence used to estimate the percentage of cells in G_1_, G_2/M_ or apoptosis (Ap) at 24 or 48 hrs following culture inoculation with or without doxycycline induction for ISceI bearing strains (red line, label on the right) in comparison with parental strain (blue line). B) Growth analysis of strains is shown over 5 days of consistent cell passage with (dashed lines) and without (solid lines) doxycycline induction for 70.II-ISceI (●), Δ70-ISCEI (▲), Dimer- ISceI (◆), Dimer_Rv-ISceI (■), and Mut_Dimer-ISceI(▾).
(PDF)

S1 Dataset Raw files of BES 70-bp repeats from *Lister427* & *TREU927*. FASTA-formatted input sequences of 70-bp repeats used to make individual sequence logos (weblogo.berkely.edu). Length differences are shown as dashes.
(PDF)

S1 Table Table of minichromosomal *VSG* contings containing 70-bp the repeat consensus. Minichromosomal DNA was isolated by gel fractionation, deep sequenced and contigs assembled. Table shows contigs containing *VSG* name and distance to the first upstream 70-bp repeat.
(PDF)

S2 Table 70.II-ISceI switched clones. Assigned clone numbers for each switched clone arising from DSB induction in the 70.II-ISceI line are shown alongside their population number (from 5 total), determined switching mechanism, the *Lister427* number of the newly expressed *VSG* and its predicted location of genomic origin.
(PDF)

S3 Table 70.I-ISceI switched clones. Assigned clone numbers for each switched clone arising from DSB induction in the 70.I-ISceI line are shown alongside their population number (from 5 total), determined switching mechanism, the *Lister427* number of the newly expressed *VSG* and its predicted location of genomic origin.
(PDF)

S4 Table Δ70-ISceI switched clones. Assigned clone numbers for each switched clone arising from DSB induction in the 70.II-ISceI line are shown alongside their population number (from 5 total), determined switching mechanism, the *Lister427* number of the newly expressed *VSG* and its predicted location of genomic origin.
(PDF)

S5 Table VSG-Seq data table. *VSG* gene numbers (*Lister427*) for all *VSGs* selected from these experiments are shown along site their corresponding genomic locations and their proportion within the population as determined by VSG-seq for three replicates of each line analyzed (Δ70-ISceI, Dimer and Dimer_Rv).
(PDF)

S1 Text Oligos used in strain constructions. Red Nucleotides Show BES1 homology. Long oligos used to make repeats are shown as Monomer, Dimer, or Mut_Dimer. Only forward oligo shown, KpnI sites were used to clong repeat fragments into Δ70-ISceI landscape.
(PDF)
