# Supplementary figures and images for: A Conserved DNA Repeat Promotes Selection of a Diverse Repertoire of Trypanosoma brucei Surface Antigens from the Genomic Archive
Source: PLoS Genet. 2016 May 5;12(5):e1005994. doi: 10.1371/journal.pgen.1005994 (PMC4858185; doi:10.1371/journal.pgen.1005994)

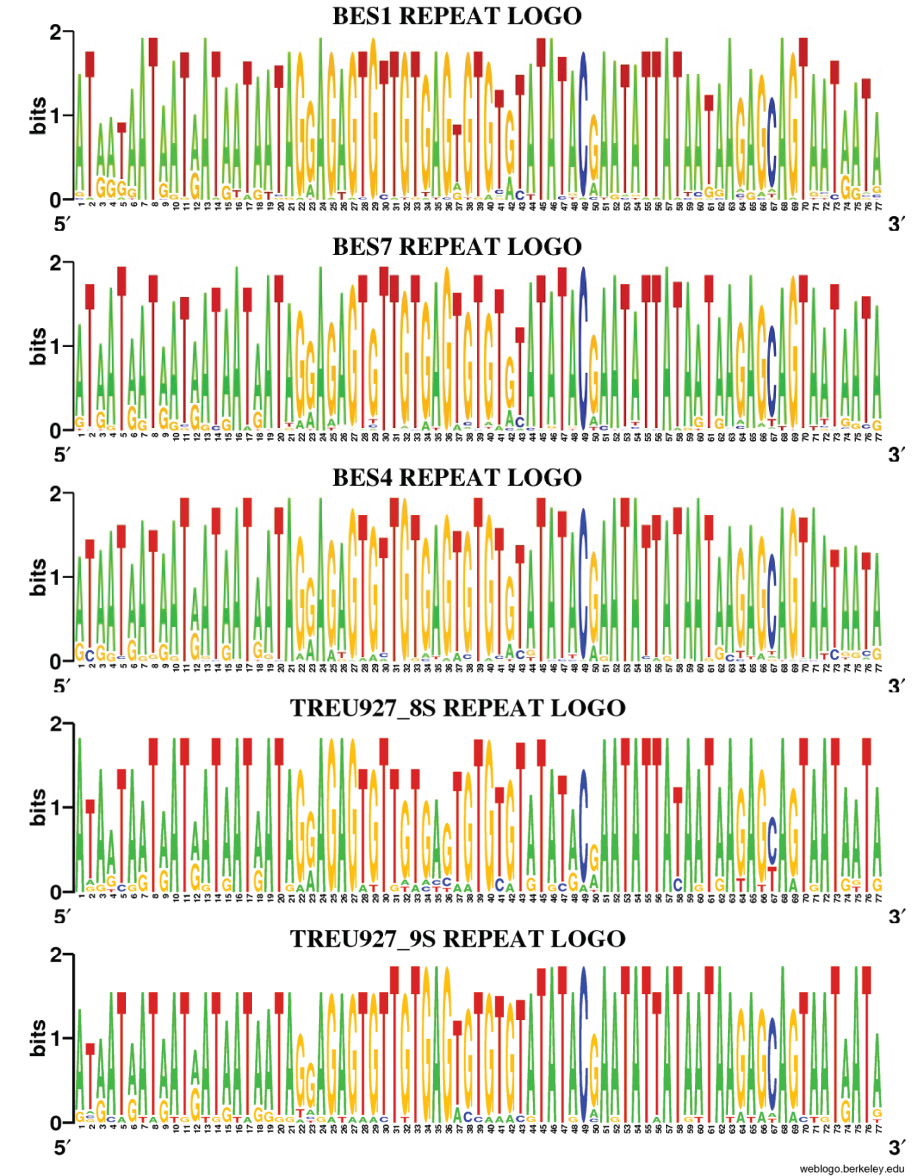

Supplement: S1 Fig — The consensus DNA repeat is shown as a logo [weblogo.berkely.edu] produced from the repeat motifs in three BESs from Lister427 and two BESs from TREU927. (JPG) [file pgen.1005994.s001.jpg]

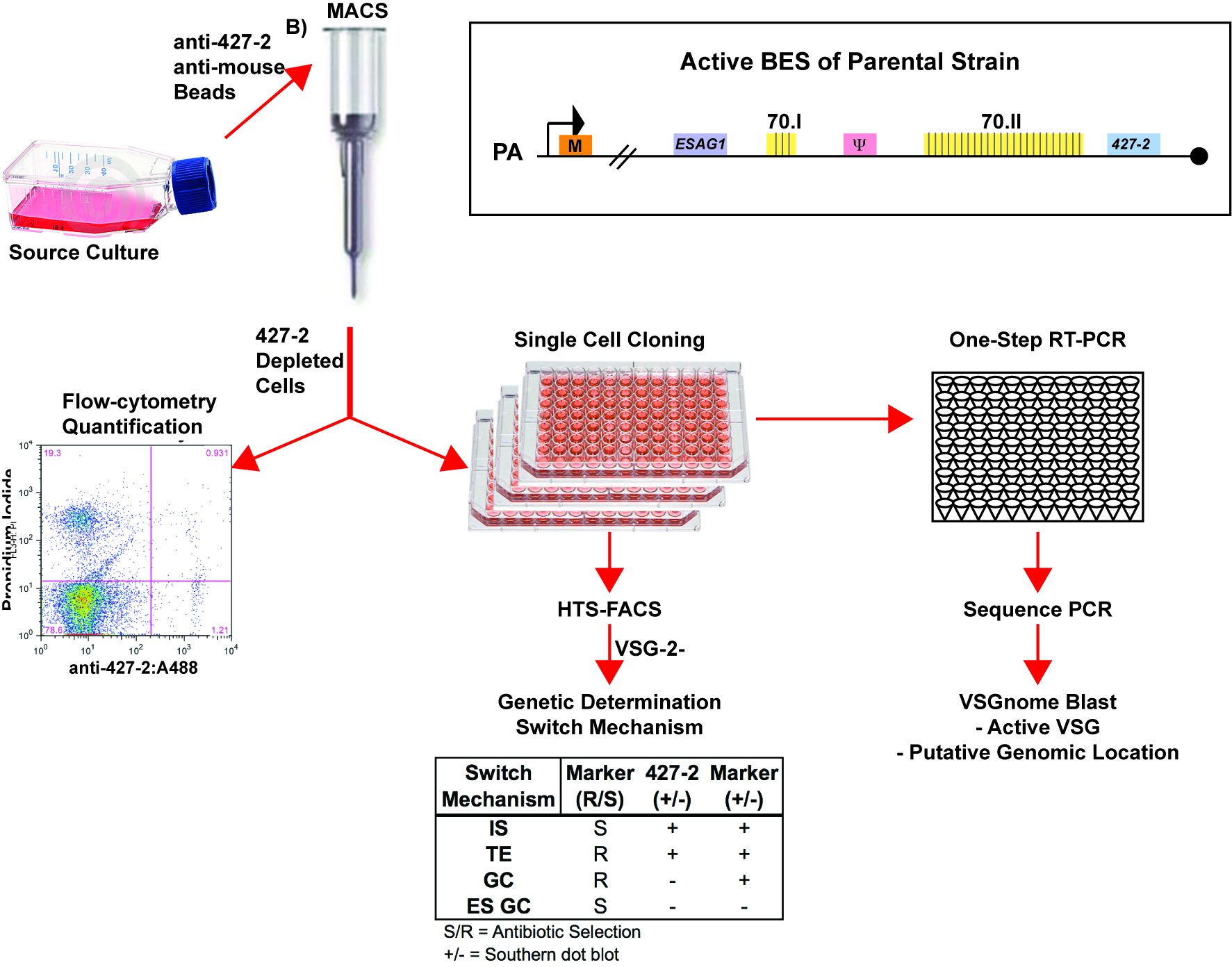

Supplement: S2 Fig — Cultures grown for switching analysis are depleted of cells harboring the original VSG coat by MACS analysis were split determine switching frequency by flow-cytometry (as described previously) and for single cell cloning. Switched clones were validated by high-throughput FACS (HTS-FACS) analysis and their mechanisms of switching determined using a set of genetic criteria. RNA was extracted from each clone and subjected to One-step RT-PCR followed by direct PCR sequencing and VSGnome Blast of the PCR products. (TIF) [file pgen.1005994.s002.tif]

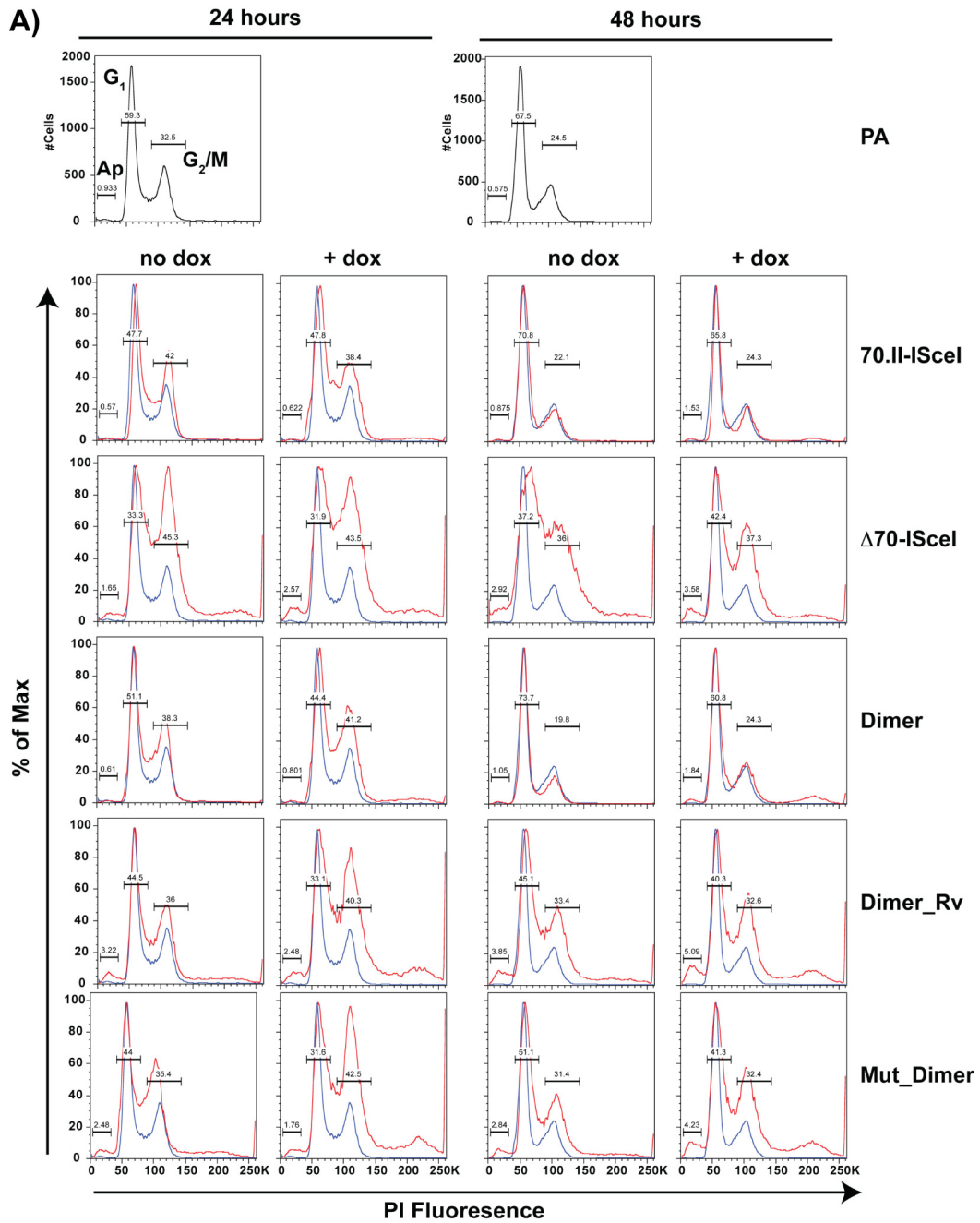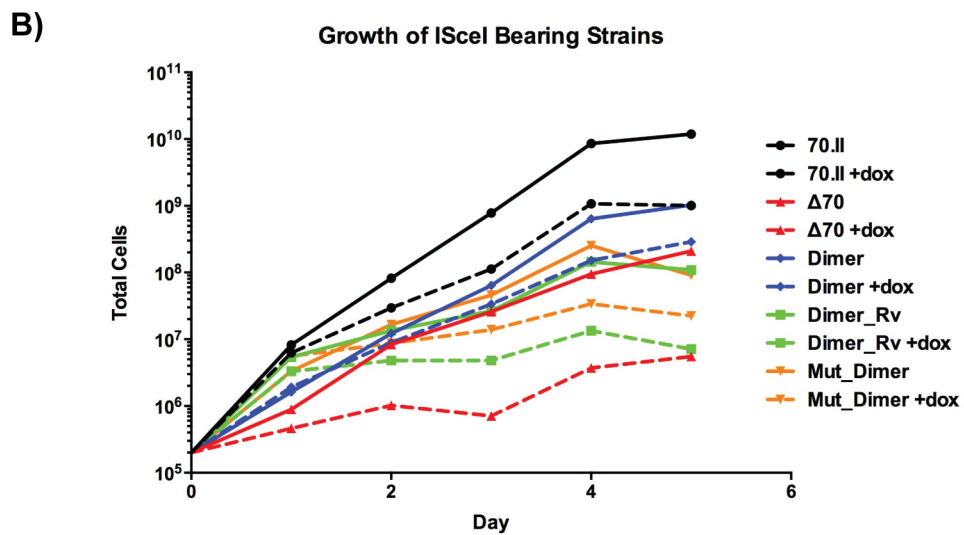

Supplement: S3 Fig — A) DNA content frequency histogram measured by PI fluorescence used to estimate the percentage of cells in G1, G2/M or apoptosis (Ap) at 24 or 48 hrs following culture inoculation with or without doxycycline induction for ISceI bearing strains (red line, label on the right) in comparison with parental strain (blue line). B) Growth analysis of strains is shown over 5 days of consistent cell passage with (dashed lines) and without (solid lines) doxycycline induction for 70.II-ISceI (●), Δ70-ISCEI (▲), Dimer- ISceI (◆), Dimer_Rv-ISceI (■), and Mut_Dimer-ISceI(▾). (PDF) [file pgen.1005994.s003.pdf]
