# Supplementary material for: A Conserved DNA Repeat Promotes Selection of a Diverse Repertoire of Trypanosoma brucei Surface Antigens from the Genomic Archive
Source: PLoS Genet. 2016 May 5;12(5):e1005994. doi: 10.1371/journal.pgen.1005994 (PMC4858185; doi:10.1371/journal.pgen.1005994)
Supplement: S1 Dataset — FASTA-formatted input sequences of 70-bp repeats used to make individual sequence logos (weblogo.berkely.edu). Length differences are shown as dashes. (PDF) [file pgen.1005994.s004.pdf]

Lister427 BES1 Repeats FASTA Files (70.I & 70.II)

```
>70.1-73
ATGATAATAGTAATGATAATAGGAGAGTGTATGTTGGTTAGATTTA--CAAAAGTTATTCTCAAAAAA-
TAATGATA
>70.1-132
CAAAAAATAATGATAATAATAGGAGTGTGTTGTGAGTGTGAGTATATACGAATATTATAATAAGAGCAGT
AATAATA
>70.1-218
ATGATAATAATAATAATAATAGAAGAGTGTTGTGAGTGTGTATATA--
CAAATATTATAATAAGAGCAGTAGCGATA
>70.1-401
ATAATAATAATAATAATAATAGGAGAGTGTTGTGAGTGTGCATATA--
CGAATATTATAATGACAGCAGTTTTAGAC
>70.2-224
ATAATAATGATAATAATAATAGGAGAGTGTTGTGAGTGTGTGTATATACGAATATTATAATAAGGGCAGT
AATAATA
>70.2-316
ATGATAATGATAATAATAATAGGAGAGTGTTGTGAGTGTGTGTATATACGAATATTATAATAAGAGCAGT
AATAATA
>70.2-411
ATGATAATAATAATAGTGATAGTAGAGTGTTGTAAGTGTGTGTATATACGAATATTATAATAAGGGCAGT
AATAATA
>70.2-503
ATGATAATGATAATAATAATAGGAGAGTGTTGTGAGAGTG--
TATATACGAATATTATAATAAGAGCAGTAATAATA
>70.2-598
ATTATAATAA-
AATAATAATAGAAGAGTGTTGTGAGTGTGTGTATATACAAATATTATAATAAGAGCAGTAATAATA
>70.2-672
ATAATAATAATGATATTATTAGAAGAGTGTTGTGAGTGTGT--
ATATACGAATATTATAATAAGAGCAGTGATAATA
>70.2-768
ATAATAATAA-
AATAATAATAGAAGAGTGTTGTGAGTGTGTGTATATACGAATATTATAATAAGAGCAGTGATAATA
>70.2-850
ATGATAATAATAATAATAATAGAAGAGCGTTGTGAGTGTGT--
ATATACGAATATTATAAGAAGAGCAATAATAATA
>70.2-931
ATGATAATAATAATAAAAATAGGAGAGTGTTGTGAGTGTGTGTATATACGAATATTATAAGAAGAGCAGT
AATAATA
>70.2-1014
ATAATAATAATAATAATAATGGGAGAGTGTTGTGAGTGTGTGTATATACGAATATTATAAGAAGAGCAGT
AATAATA
>70.2-1097
ATGATAATAATAATAATAATAGGAGAGTGTTGTGACTGTGTATATA--
CGAATATTATAAGAAGAGGAGTAATAATA
>70.2-1178
ATGATAATAATAATAAAAATAGGAGAGTGTTGTGAGTGTGTGTATATACGAATATTATAAGAAGAGCAGT
AATAGTA
```

>70.2-1264
ATAATAATAATGATATTAATAGGAGAGTGTTGTGAGTGTGTGCATATACGAACATTATAATAAGAGCAGT
AATAATA
>70.2-1338
ATAATAATAATAATAATAAGAGGAGAGTGTTGTGAGTGTGCGTATATACCAGTATTATAATAAGAGCAGT
AATAATA
>70.2-1424
ATAGTAATAATAATATTAATAGGAGAATGTTGTGAGTGTGTGCATATACGAATATTATAATAAGAGAAGT
AATAATA
>70.2-1501
GTAAAGATGATAATGATAATAGGAGTGTGTTGTGAGTGTATGTATATACGAATATTATAATAAGAGCAGT
AATAATG
>70.2-1605
ATGATGATAATGAAAATAATAGGAGTGTGTTGTGAGTGTGTATATA--
CGAATATTATAATAAGAGCAGTAATAATG
>70.2-1695
ATGATAATAATAATAATAATAGGAGAGTGTTGTGAGAGTGT--
ATATACGAATATTATAATAAGAGCAGTAATAATA
>70.2-1773
ATGATAATAATAATA------GGAAAGTGTTGTGAGTGTGT--
ATATACGAATATTATAATAAGAGCAGTAATAATA
>70.2-1839
ATAGTAATAATAATAATGATAGGAGAGTGTTGTGAGTGTGTGTATACTAATATTATAATGA--
GAGCAGTAATAATA
>70.2-1911
ATAGTAATGATAATAATAATAGGAGAGTGTTGTGAGAGGGTGTATATACAAATATTATAATAAGAGCAGT
AATGATA
>70.2-2015
ATAGGAATAATGATAATAATAGGAGAGTGTTGTGAGTGTGT------
ACGAATATTATAATAAGAGCAGTAATGATA
>70.2-2107
ATAATAATAGGAATAATAATAGGAGAGTGTTGTGAGGGTGTGTATATACGAATATTATAATAAGAGCAGT
AATGATA
>70.2-2196
ATAGGAATAATGATAATAATAGGAGAGTGTTGTGAGTGTGT------
ACGAATATTATAATAAGAGCAGTAATGATA
>70.2-2279
ATAGGAATAATGATAATAATAGGAGAGTGTTGTGAGGGTGTGTATATACGAATATTATCATGAGAGCAGT
AATAGTA
>70.2-2365
ATAGGAATAATGATAATAATAGGAGAGTGTTGTGAGTGTGT------
ACGAATATTATAATAAGAGCAGTAATGATA
>70.2-2448
ATAGGAATAATGATAATAATAGGAGAGTGCTGTGAGGGTGTGTATATACGAATATTATAATGAGAGCAGT
AATAGTA
>70.2-2528
ATAATAATAATAATGATAATAAGAGAGTGCTGTGAGAGTGTGTATATACGAATATTATAATAAGAGCAGT
AATGATA
>70.2-2617
ATAGGAATAATGATAATAATAGGAGAGTGTTGTGAGTGTGT------

ACGAATATTATAATAAGAGCAGTAATGATA
>70.2-2784
ATAGGAATAATGATAATAATAGGAGAGTGTTGTGAGGGTGTGTATATACAAATATTATAATAAGAGCAGT
AATGATA
>70.2-2873
ATAATGATAATGATGATAATAGGAGAGTGTTGTGAGAGTGTATATATGCGAGTATTATAATAAGAGTAGT
AATGATA
>70.2-2962
ATAATAATAATAATAATGATAGGAGAGTGTTGTGAGTGTGTGTATATACGAATATTATAATAAGAGCAGT
AATAATA
>70.2-3039
ATAATAATGATAATGATAATAGAAGAGTGTTGTTAGTGTGTATATA--
CGAATATTATAATAAGAGCAGTAATAATG
>70.2-3108
ATAATGATAATGATAATATCAGGAGAGTGTTGTGAGTGTGTATATA--
CGAATATAATAATAAAAGCAGTAATAATA
>70.2-3186
ATAATAATAATGATAATGATAGGAGAGTGTTGTGAGTGTGTGTATATACGAATATTATAATAACACCAGT
AATAGTA
>70.2-3269
ATAAGAATCATAATGATAATAGGAGAGTGTTGTGAGTGT--
ATATATACAAATATTATAATAAGAGCAGTACTAATG
>70.2-3335
GTACTAATGATAATAATATTAGGAGAGTGTTGTGAGTGTGTGTATATACGAATATTATACTAAGAGCAGT
AATAAGT
>70.2-3406
ATAAGTATAATAATAATAATAGGAGAGTGTTGTGAGTGTGTGCTTA-
CCAATATTATAATAATGATAGTAACGACC

Lister427 BES4 Repeats FASTA Files

>BES4 REPEAT-65
ATAATAATAATGATAATGATAGAAGAGTGTTGTGAGAGTG--
TTTATACGAATATTATAATAAGAGCAGTAATAATA
>BES4 REPEAT-131
GTAATAATAATGATAATAATAGGAAAGTGTTGTGAGTGTGTGTATATACAAATATTATAATAAGAGCAGT
ACTAATA
>BES4 REPEAT-220
ATAGTAATAATAATAATAATAGGAGAGTGTTGTGAGTGTGTGTGTATATACGAATATTATAATAAGAACA
GTAATAA
>BES4 REPEAT-305
ATAATAATAATAATAGTGATAGGAAAGTGTTGTGAGTGTGTGTATATACAAATATTATAATAAGAGCAGT
AATAATA
>BES4 REPEAT-388
GTAAAAATAATGATAATGATAGGAGAGTGTTGTGAGTGTGTGTATATACAAATACTATAATAAGAGCAGT
AATAACA
>BES4 REPEAT-462
ACAATAATAATGATAATAATAGGAGAGTGTTGGGAGTGTGTATATA--
CGAATATTATAATAAGAGCAGTAATAATG

>BES4 REPEAT−525
GCAGTAATAATGATAATAATAGGAGAGTGTTGTGTGTGTGGATATA−−
CGAATATTATAATAAGAGCAGTAATAATA
>BES4 REPEAT−594
ATAATAATAATAATAATAATAGGAGAGTGTTGTGAGTGTGTGCATATACGAATATTGTAATGAGAGCAGT
ATCAATA
>BES4 REPEAT−671
ATAATAATAATGATAATGATAGAAGAGTGTTGTGAGAGTGTTTATA−−
CGAATATTATAATAAGAGCAGTAATAATA
>BES4 REPEAT−737
GTAATAATAATGATAATAATAGGAAAGTGTTGTGAGTGTGCATATA−−
CGGATATTATAATAAGAGCAGTAATAATA
>BES4 REPEAT−815
ATAATGATAATAATGATAATAGGAGTGTGTTGTGAATGTGTATATA−−
CGAATATTATAATACGAGCAGTAATAATA
>BES4 REPEAT−923
ATAATAATAATAATAATAATAGGAGAGTGCTGTGAGTGTGTGTATATACGAATATTATAATAAGAGCAGT
AATAATA
>BES4 REPEAT−1012
ATGATAATAATAATAATAATAGGAGAGTAATGTGAGTGTGTATATA−−
CGAATATTATAATGAGAGCAGTATCAATA
>BES4 REPEAT−1087
ATAATAATAATGATAATGATAGAAGAGTGTTGTGAGAGTGTTTATA−−
CGAATATTATAATAAGAGCAGTAATAATA
>BES4 REPEAT−1153
GTAATAATAATGATAATAATAGGAAAGTGTTGTGAGTGTGTGTATATACGAATATTATAATGAGAGCAGT
AACAATA
>BES4 REPEAT−1242
ATGATAATAATAATAATAATAGGAGAGAGTTGTGAGTGTGTGTATATACGAATATTATAATGAGAGCAGT
AATAATA
>BES4 REPEAT−1316
ATAATAATGATAATAATGATAGGAGAGTGTTGTGAGTGTGTATATC−−
CGAATATTATAATACGAGCAGTAATAATA
>BES4 REPEAT−1394
ATAATGATGATGATGATAATAGAAGGGTGTTGTGAGTCTGTGTATA−−
CGAATATTATAATAAGAGCAGTAATAACA
>BES4 REPEAT−1466
ACAATAATAATGATAATAATAGGAGAGTGTTGTGAGTGTG−−
TATATACGAATATTATAATGAGAGCAGTATCAATA
>BES4 REPEAT−1541
ATAATAATAATGATAATGATAGAAGAGTGTTGTGAGAGTGT−−
TTATACGAATATTATAATAAGAGCAGTAATAATA
>BES4 REPEAT−1607
GTAATAATAATGATAATAATAGGAAAGTGTTGTGAGTGTGTGTATATACGAATATTATAATGAGAGCAGT
AACAATA
>BES4 REPEAT−1696
ATGATAATAATAATAATAATAGGAGAGAGTTGTGAGTGTGTGTATATACGAATATTATAATGAGAGCAGT
AATAATA
>BES4 REPEAT−1770
ATAATAATGATAATAATGATAGGAGAGTGTTGTGAGTGTGT−−

ATATCCGAATATTATAATACGAGCAGTAATAATA
>BES4 REPEAT-1848
ATAATGATGATGATGATAATAGAAGGGTGTTGTGAGTCTGTGTATA--
CGAATATTATAATAAGAGCAGTAATAACA
>BES4 REPEAT-1920
ACAATAATAATGATAATAATAGGAGAGTGTTGGGAGTGTG--
TATATACGAATATTATAATAAGAGCAGTAATAATG
>BES4 REPEAT-1983
GCAGTAATAATGATAATAATAGGAGAGTGTTGTGTGTGTG-GA-
TATACGAATATTATAATAAGAGCAGTAATAATA
>BES4 REPEAT-2052
ATAATAATAATAATAATAATAGGAGAGTGTTGTGAGTGTGTGCATATACGAATATTGTAATAAGAGCAGT
AATAATA
>BES4 REPEAT-2123
ATAATAATAATAATAATAATAGGAGAGTGTTGTGAGTGTGTGTATATACAAATATTATAATAAGAGCAGT
AATAATA
>BES4 REPEAT-2203
ATAATAATGATAACAATAACAGGAAAGTGTTGTGAGTGTGC--
ATATACGGATATTATAATAAGAGCAGTAATAATA
>BES4 REPEAT-2281
ATAATGATAATAATGATAATAGGAGTGTGTTGTGAATGTGT--
ATATACGAATATTATAATACGAGCAGTAATAATA
>BES4 REPEAT-2389
ATAATAATAATAATAATAATAGGAGAGTGCTGTGAGTGTGTGTATATACGAATATTATAATAAGAGCAGT
AATAATA
>BES4 REPEAT-2478
ATGATAATAATAATAATAATAGGAGAGTAATGTGAGTGTG--
TATATACGAATATTATAATAAGAACAGTAATAGTA
>BES4 REPEAT-2553
ATAACAATAATAATAATTGTAGGAGAGTGTTGTGAGTGTGTGTATATACGAATATTATAATAAGAGCAGT
AATAGTA
>BES4 REPEAT-2624
ATAGTAATAATAATAATAATAGGAAAGTGTTGTGAGTGTGTGTATATACGAATATTATAATAAGAACAGT
AATAGTA
>BES4 REPEAT-2701
ATAACAATAATAATAATTGTAGGAGAGTGTTGTGAGTGTGTGTATATACGAATATTATAATAAGAGCAGT
AATAATA
>BES4 REPEAT-2931
ATAATAATAATAATAATGATAGGAGAGTGTTGTAAGTGTGTGT--
ATACAAATATAATAATAATAGTAGTAATAATA
>BES4 REPEAT-3006
GTAATGATGATGGTGATAATAGGAGAGTGTTGTGAGTATGTGTATA--
CAAATATAATAATAAGAGTAGTAACAATA
>BES4 REPEAT-3090
ATGATAATAATAATAATAATAGGAGAGTGTTGTGAGTGTGTATATATACAAATATTATAATAATAGGAGT
AATTATG
>BES4 REPEAT-3179
ATAATGATAATGATAATAATAGAAGAGTGTTGTGAGTGTGTGTATATACGAATATTATAATAAGAGCAGT
AATAATA
>BES4 REPEAT-3256

ATAATGATAATGATAATAATAGAAGAGTGTTGTGAGTGTGTGTATATACGAATATTATAATAAGAGCAGT
AATAATA
>BES4 REPEAT-3333
ATAATGATAATGATAATAATAGAAGAGTGTTGTGAGTGTGTGTATATACGAATATTATAATAAGAGCAGC
AATGACA
>BES4 REPEAT-3413
ATAATAATAATGCTAATAATAGAAGAGTATTGTGAGTGTATGTATA--
CGAATATTATAATAAGAGCAGTAATAATA
>BES4 REPEAT-3494
ATAATAGGAATAATGATAATAAGAGAGTGTTGTCAGTGTGTGCATATACGAATATTATAATAAGAGCAGT
AATATTA
>BES4 REPEAT-3571
ATAGTAATGATGATAATAATAGAAGAGTGTTGTGAGTGTGTGTGTATACGAATATTATAATAACAGCAGT
AATAATA
>BES4 REPEAT-3636
GCAGTAATAATAATAATAATAGAAGAATGTTGTGAGTGTGTGTGTATACGAATATTATAATAAGGCCAGT
AATAATG
>BES4 REPEAT-3707
ATAATGATGATGATAATGATAGGAGAGTGTTGTGAGTGTGT--
ATATACGAATATTATAATAAGAGCAGTAATAATG
>BES4 REPEAT-3785
ATGATGATAATAATAATAATAAGAGAGTATTGTGAGTGTG--
TATATACGAATACTATAATAAGAGCAGTAATAATA
>BES4 REPEAT-3866
ATGATAATAATAATAATAATAGAAGAGTGTTGTGGGTGTGT--
ATATACGGATATTATAAGAAGAGCAGTAATAATA
>BES4 REPEAT-3947
ATGATGATGATAATAATAATAGAAGAGTGTTGTGAGTGTGT--
ATATACGAATATTATAATAATAGTTGTAATAATA
>BES4 REPEAT-4016
ATAATAATAATAATAATAATAGAAGAGTGTTGTGAGTGTGTGTTTATT--
AATATTATAATAAGAGCAGTAATAATA
>BES4 REPEAT-4085
ATAATAATAATTATAATAATAGGAGAGTGTTGTGAGTGTATGTATA--
CGAATATTATAATAAGAGCAGTAATAATA
>BES4 REPEAT-4166
ATAATAGGAATAATGATAATAAGAGAGTGCTGTAAGAGTGTGTATATACGAATATTATAATAACAGCAGT
AATAAAA
>BES4 REPEAT-4249
ATAATAATGATGATAATAATAGAAGAGTGTTGTGAGTGTGTATATATACAAATATTATAATAATAGGAGT
AATTATG
>BES4 REPEAT-4338
ATAATAGGAATAATGATAATAAGAGTGTGTTGTCAGTGTGTGCATATACGAATATTATAATAAGAGCAGT
AACAATG
>BES4 REPEAT-4409
ACAATGATAATAATAATAATAGGAGTGTGTTGTAAGTGTGTGTATATACGAATATTGTAATGAGAGCAGT
AATAAAA
>BES4 REPEAT-4477
GTAATAAAAATGATAATAATAGGAGAGTGTTGTGAGTGTGTGTATA--
CGAATATTATAATAAGAAAAATAATAATA

```
>BES4 REPEAT-4579
ATGATGATAATAATAATGATAGGAGAGTGTTGTGAGTGTGTGTATATACGAATATTATAATAAGAGCAGT
AATAATA
>BES4 REPEAT-4647
GTAATAATAGTAATAATAATAGGAGAGTGTTGTGAGTGTGTATATA--
CGAATATTATAATAAGAGCAGTAATAATA
>BES4 REPEAT-4713
GTAATAATAGTAATAATAATAGGAGAGTGTTGTGAGTGTGT--
ATATACGAATATTATAATAAGTGCAGTAATAGTA
>BES4 REPEAT-4797
ATAATAATAATAA-AATGGTATCAGAGTGTTGTGAGTGTGTGCATA--
CGAATATTATAATAAGAGCAGTAATAATG
>BES4 REPEAT-4916
ATGATGATAATAATAATGATAGGAGAGTGTTGTGAGTGTGTGTGTATACGAATATTATAATGAGAGCAGT
AATAATA
>BES4 REPEAT-4999
ATAATAATGGTAGTGATATTAGGAGAGTGTTGTGAGTGTGTGTATA--
CGAATATTATAATAAGAGCAGTAATGATA
>BES4 REPEAT-5071
ATAATAATAATAATGATGATAGGAGAGTGTTGTGAGTCTG--
TATATACAAATATTATAATAAGAGCAGTAATGATA
>BES4 REPEAT-5143
ATAATAATAATAATAATAATAGAAGAGTGTTGTGAGTGTG--
TATATACGAATATTATAATAAGAGCAGTAATACTA
>BES4 REPEAT-5224
ATAATGATAATAATAATAATAGGAGAGTGTTGTGAGTGTGTGTGTACTAAAATAATAAGAGTATTAAAGA
CTACAGT
```

Lister427 BES7 Repeats FASTA Files

```
>BES7 REPEAT-62
ATAATAATAATAATAATAATGCGAAAGTGTTGTGAGTGTGTGTATATACGAATTTTATAGTAAGAGCAGT
AATAACA
>BES7 REPEAT-154
ATAATAATAATAATAATAATAGGAGAGTGTTGTGAGTGTGTGTATATACGAATTTTATAGTAAGAGCAGT
AATAACA
>BES7 REPEAT-237
ATAATAATAATAATAATAATAGGAGAGTGTTGTGAGTGTGTGCATATACGAATATTATAATACGAGCAGT
AATAATA
>BES7 REPEAT-317
ATAATAATAATAATAATAATAGGAAAGTATTGTGAGTGTGTGTATATACGAATATTATAATAATAGCAGT
AACAATA
>BES7 REPEAT-394
ATTATAATGATAATAATAATAGAAGAGTGTTGCGAGTGTGTATATA--
CGAATATTATAAGAAGAGCAGTAATAGTA
>BES7 REPEAT-463
ATAGTAATAATAATAATGATAGGAAAGTGTTGTGAGTGTGTATATA--
CAAATATTATAATAAGAGCAGTAATAATA
>BES7 REPEAT-529
```

GTAATAATAATAATAATAATAAAAGAGTTTTGTGAGTGTGTCCATATACGAATATTATAATGAGAGCAGT
AATAATA
>BES7 REPEAT-606
GTAATAATAATAATAATAATAAAAGAGTGTTGTGAGTGTGTATATA--
CGAATATTATAATAAGAGTAGTAATAATA
>BES7 REPEAT-681
ATAATGATAATAATAATAATAGGAGAGTTTTGTGAGTGTCTGTATATACTAATATTATAATAAGAGTAGT
AATAATA
>BES7 REPEAT-764
ATAATAATAATGATAATAATAGGAGAGTTTTGTGAGTGTG--
TATATACGAATATTATAATAAAAGTAGTAATAATA
>BES7 REPEAT-845
ATAATAATAATAATAATAATAAGAAAGTGTGTTGTGAGTGTGTATATACGAATATTATAATAAGAGCAGT
AAAAATA
>BES7 REPEAT-925
ATGATGATGGTAATAATAATAGGAGCGTGTTGTGAGTGTGTATATA--
CGAATATTATAATAAGAGCAGTATTAATA
>BES7 REPEAT-1003
ATAATAGTAATGACAATGATAGAAGAGTGTTGTGAGAGTGTGTATA--
CAAATATTATAATAAGAGCAGTAATAATA
>BES7 REPEAT-1084
ATGATAATAATAATAATAATAGAATAGTCTTGTGAGTGTGTATATA--
CGAATATTATAATAAGAGCAGTAATAATG
>BES7 REPEAT-1153
ATAATGATAATAATAATGATAGGAGAGTGTTGTGAGTGTGTGTATATACGAATATTATAATAAGAGCAGT
ATTAATA
>BES7 REPEAT-1233
ATAATAGTAATGACAATGATAGAAGAGTGTTGTGAGTGTGTGTATATACGAATATTATAATGAGAGCAGT
AATAATA
>BES7 REPEAT-1319
ATAATGATGATAATAATAATAGGAGAGTGTTGTGAGTGTGTGTATATACGAATATTATAATAAGAGCAGT
ATTAATA
>BES7 REPEAT-1390
TTAATAATAATAATAATGATAGGAGAGTGTTGTGAGTGTATGTATAT--
GAATATTATAATAAGAGCAGTTATAATA
>BES7 REPEAT-1462
ATAATAATGATGATAATGATAGAAGAGTGTTGTGAGTGTGTGTATATACGAATATTATAATGAGAGCAGT
AATAATA
>BES7 REPEAT-1545
AAGATAATGATGATAATAATAGAATAGTCTTGTGAGTGTGTATATA--
CGAATATTATAATAAGAGCAGTAATAATG
>BES7 REPEAT-1614
ATAATGATAATAATAATGATAGGAGAGTGTTGTGAGTGTGTGTATATACGAATATTATAATAAGAGCAGT
ATTAATA
>BES7 REPEAT-1694
ATAATAGTAATGACAATGATAGAAGAGTGTTGTGAGTGTGTGTATATACGAATATTATAATGAGAGCAGT
AATAATA
>BES7 REPEAT-1780
ATAATGATGATAATAATAATAGGAGAGTGTTGTGAGTGTGTATATA--
CGAATATTATAATGAGAGCAGTAGTAATA

>BES7 REPEAT-1984
GTAATAATCATAATAATGATAGGAGAATGTTGTGAGTGTGTGTATA--
CGAATATTATAATAAGATCAGTAATAATA
>BES7 REPEAT-2065
GTGATGATAATAATGATAATAGGAGAGTGTTGTGAGTGTGTGCATATACGAATCTTATAATAAGAGCTGT
ACTAATA
>BES7 REPEAT-2148
ATAATAATGGTAATAATAATAGGAGAGTGTTGTGAGTGTGTGCATATACGAATATTATATTAAGAGCAGT
AATAATA
>BES7 REPEAT-2231
GTAATAATAATAATAATGATAGGAAAGTGTTGTGAGTGTGTGTATATACGAATATTATAATAAGAGCAGT
AATAATA
>BES7 REPEAT-2311
ATGATAATAGCAGTAATAATAGGAGAGTGTTGTGAGTGTGTGTATA--
CAAATATTATAATAAGAGTAGTAATAATA
>BES7 REPEAT-2389
ATGATAATAGCAGTAATAATAGGAGAGTGTTGTGAGTGTGTGTATATACGAATATTATAATGACAGCTGT
ACTAATG
>BES7 REPEAT-2478
ATGATGATAATAATGATAATAGGAGAGTGTTGTGAGTGTGTGCATATACGAATCTTATAATAAGAGCAGT
AATAATA
>BES7 REPEAT-2564
ATGATAATAATGATAATAATAGGAGAGTGTTGTGAGTGTGTG--
TATACGAATATTATAATAAAAGCAGTAATGATA
>BES7 REPEAT-2645
ATGATAATAATAATAATAATAAGAGAGTGTTGTGAGTCTGTATCTG--
CGAATATTATAGTAAGAGCAGTAATCGTA
>BES7 REPEAT-2729
GTAATAATAATGATAATTATTGGAGAGTGTTGTGAGTCTGTATCTG--
CGAATATTATAGTAAGAGCAGTAATCGTA
>BES7 REPEAT-2813
GTAATAATAATGATAATTATTGGAGAGTGTTGTGAGTGTGTA------
CGAATATTATAATAAGACCAGTAATAATA
>BES7 REPEAT-2881
ATAATAATGATAATAATATAAGGATATTGTTGTGAGAGTGTGAATATACGAATATTATAATAACAGCAGT
AATAATA
>BES7 REPEAT-2957
ATAATAATAATGGTAATAATAGAAGAGTGTTGTGAGTGTGAGTATATACGAATATTATAATAAGAGCAGT
AATAATA
>BES7 REPEAT-3046
ATAATAATAATAATAATAATAGGAGAGTGTTGTGAGTGTGTGTATA--
CGAACATTATAGTAAGAGCAGTAATAATA
>BES7 REPEAT-3130
ATAATAATAGTAATAATAATAGGAGAGTGTTGTGA-
TATGTGTATATACGAATTTTAAAATTAGAGGAGTAATAATA
>BES7 REPEAT-3206
ATAGTAATGATGGTAATAATAGGAGAGTGTTGTGAGTGTGAGTATATACGAATATTATAATGAGAGCAGT
AATAATA
>BES7 REPEAT-3286
TTTATAATAATGATAATAATAGAAGGGTGTTGTGTGTGTGAGTATATACGAATATTATAATAAGAGCAGT

AATAATG
>BES7 REPEAT−3372
ATAATAGGAATGATAATAATAGAAGAGTGTTGTGAGTGTGTGTATA−−
CGAATATTATAATAAGAGCAGTAATAATG
>BES7 REPEAT−3453
ATAATAATAGTAATGATAATAGGAGAGTTTTGTGAGAATGT−−
ATATACGAATATTATAATAAGAGCAGGAATAATA
>BES7 REPEAT−3540
ATAATAATGATGATAATAATAGGAGAGTCTTGTGAGTGTGTATAT−−
GCGAATATTATAATAAGAGCAGTAATAATA
>BES7 REPEAT−3612
ATAATAATAATAATAATAATAGGAGAGTGTTGTGAGTGTGT−−
ATATACGAATATTATAATAAGAGCAGTAATAATA
>BES7 REPEAT−3693
ATAATGATGATAATAATAATAGGAGTGTGTTGTAAGTGTGT−−
ATATACGAATATTATAATAAGAGCAGTAATAATA
>BES7 REPEAT−3765
ATAATAATAATAATGATAATGGGAGAGTGTTGTGAGTGTGT−−
ATATACGAATGTTATAATAAGAGCAGTAATAATA
>BES7 REPEAT−3843
ATGATAGTAATGATGATAATAGGAGAGTGTTGTGAGTGTGTGTATA−−
CGAATATTATAATAAGAGTAGTAATGATA
>BES7 REPEAT−3927
ATAATGATAATGATAATAATAGGAGAGTGTTGTGAGTGCG−−
TATATACGAATATTATAATAAGAGTAGTAATGATA
>BES7 REPEAT−4005
ATAATAATAATGATAATAATTGGAAAGTGTTGTGAGTGTGTGTATATACGAATATTATAATAAGAGCAGT
AATAACA
>BES7 REPEAT−4094
ATAATGATAATAATAATAATAGAAGAGTGTTGTGAGTGTGTATATATACTAATATTATAATAAGAGCAGT
AATAATA
>BES7 REPEAT−4180
ATAATGATAATAATAATAATAGGAGAGTGTTGTGAGTTTGTATATATACGAATATTATAATAGGAGCAGT
AATAATA
>BES7 REPEAT−4263
ATAATAATAAGGATAATAATAGGAGAGTGTTGTGAGTGTATGTATATACTAATATTATAATAAGAGCAGT
TATAATA
>BES7 REPEAT−4352
ATAATAGTAATAATAATGATAGGAGAGTGTTGTGAGTGTGTATATA−−
CAAATATTATAATGAGAGCAGTAATAATA
>BES7 REPEAT−4436
ATGATGATAATAATAATAATAGGAGAGAGTTGTGAGTGTGT−−
ATATACGAATATTATAATAAGAGCAGTAGTAATG
>BES7 REPEAT−4517
ATGATGATGATAATAATGATAGGAGAGTGTTATGAGTGTGTGTATATACGAATATTATAATAAGAGCAGT
AATAATA
>BES7 REPEAT−4600
ATAATAATAAGGATAATAATAGGAGAGTGTTGTGAGTGTATGTATATACTAATATTATAATAAGAGCAGT
TATAATA
>BES7 REPEAT−4689

ATAATAGTAATAATAATGATAGGAGAGTGTTATGAGTGTGTGTATATACGAATATTATAATAAGAACAGT
AATAATA
>BES7 REPEAT-4769
ATGATGATAATAATAATAATAATAGAGTGTTGTGAGTGTTTCCATATACGAATATTATAATAAGAGCAGT
AATAATA
>BES7 REPEAT-4864
ATAATGATGATGATGATAATAGGAGAGTGTTGTGAGTGT----
ATATACGAATATTATAATAACAGAAGTAATTATA
>BES7 REPEAT-4961
ATAATAATAATAATAATAATAGGAGAGTGTTGTGAGTGTGT--
ATATACGAATATTATAATAAGAGCAGTAATAGTA
>BES7 REPEAT-5036
ATAATAATGATAATGATAATAGAAGAGTGTTGTGAGTGTGT--
ATATACGCATATTATAATAAGAGCAGTAATAATA
>BES7 REPEAT-5099
GCAGTAATAATAATGATAATAGGAGAGTGTTGTGAGTGTGTGTATATACGAATATTATGATAAGAGCAGT
AATGATA
>BES7 REPEAT-5176
ATAATAATAATAATAATGATAGGAGAGTGTTGTGAGTGTGT--
ATATACGCATATTATAATAAGAACAGTAATAATA
>BES7 REPEAT-5254
ATGATGATAATAATAATAATAATAGAGTGTTGTGAGTGTTTCCATATACGAATATTATAATAAGAGCAGT
AATAATA
>BES7 REPEAT-5349
ATAATGATGATAATAATAATAGGAGAGTGTTGTAAGTGTGTGTATATACGAATATTATAATAAGAGCAGT
AATGATA
>BES7 REPEAT-5444
ATAATGATAATGATAATAATAGGAGAGTGTTGTGAGTGTGTGTATATACGAATATTATAATAAAAGCAGT
AATAATA
>BES7 REPEAT-5521
ATAATAATGATAATAATGATAGGAGAGTGTTGTGAGTGTG--
TATATACGAATATTATAATAAGAGCAGTAATAATA
>BES7 REPEAT-5602
ATAATAATGATAATAAAAATAGGAGAGTGTTGTAAGTGTGTGTATATACAAATATTATAGTAAGAGCAGT
AATAATA
>BES7 REPEAT-5673
ATAATAATGATAATAAAAATAGGAGAGTGTTGTAAGTGTGTGTATATACAAATATTATAGTAAGAGCAGT
AATAATA
>BES7 REPEAT-5744
ATAATAATGATAATAAAAATAGGAGAGTGTTGTAAGTGTGTGTATATACAAATATTATAGTAAGAGCAGT
AATAATA
>BES7 REPEAT-5815
ATAATAATGATAATAATAATAGGAGAGTGTTGTAAGTGTGTGTATATACGAATATTATAATAAGAGCAGT
AATGATA
>BES7 REPEAT-5910
ATGATGATAATGATAATAATAGGAGAGTATTGTG-GTCT---
AAATTTCACTAAACAAGTTAAAGAATTTATTGCAG

TRU927 BES8S Repeats FASTA Files

>TREU927_8S_#1
AAAGTAATGATGATAATAATAGGAGAGTGTTGTGAGTGTGTGTATGTGCGAATATTACAATGAGAGCAGT
AATAATA
>TREU927_8S_#2
ATAATAATAATAATAATAATAGAAGAGTGTTGTGAGTGTGTGTATACACGAATATTACAATGAGAGCAGT
AATAATA
>TREU927_8S_#3
ATAGTAATAATAATAATGATAGGAGAGTGTTGTGAGTGTGTATATATGCAAATATTATAATAAGAGCAGT
AATAATA
>TREU927_8S_#4
ATAATAATAATAATAATAATAGGAGAGTGTTGTGAGTGTGTGTATATGA--
ATATTATAATAAGAGCAGTAATATTA
>TREU927_8S_#5
ATATTAGTAATGATGATAATAGGAGAGTTTTAAGA----
GTGTATATACAAATATTATAATAAGAGCAGTAATAATA
>TREU927_8S_#6
ATAATAATAATAATAATGATAGGAGAGTGTTGTGCGTATGC--
ATATACGAATATTATAATAAGAGCAGTAATGATA
>TREU927_8S_#7
AGAGCAGTAATGATAATAATAGGAGAGAGTGTTGTCAGTGTGTGTATACGAATATTATAATAAGAGTAGT
AATAGTA
>TREU927_8S_#8
ATAATAATAATGATAATAATAGGAGAGTGTTGTGAGTGTGTGT--
ATACGAATATTATAATAAGAGTAGTAATGATA
>TREU927_8S_#9
ATAATAATGATAGTAATAATAGAAGAGTGTTGTGAGTGTGT--
ATATACGAATATTATAATAAGAGCAATAATAATG
>TREU927_8S_#10
ATAATAATGATAATGATAATAGGAGAGTGTTGTGAGTGTGTGTGTATACAAATATTATAGTAAGATTAGT
GATAATG
>TREU927_8S_#11
ATGATGATGATGATAATGATGGAAGAGTGTTGTGAGTGTGCG--
TATACGAATATTATAATAAGAGCAGTAATAATA
>TREU927_8S_#12
ATAATAATAATAATAATAATAAGAGAGTGTTGTAATTGT-T-
TATATACTAATATTATAATAAGAGCAGTAATAATA
>TREU927_8S_#13
ATAATAATAATGATAATGATAGGAGAGTGTTGTCAGTGTGTGTATA--
CAAATATTATAATAATAGTAGTAATAATA
>TREU927_8S_#14
ATAATAGTAATAATAATAATAGAAGAGTGTTGTGACTGTGTGTATA--
CGAATATTATAATAATAGTAATAATAATA

TRU927 BES9S Repeats FASTA Files

>TREU927_9S_#1
ATAATGATAATAATAATAATAG-AG--TGTTGTGAGTGTGT--
ATATACGAATATTATAATAAGAGCAGTAATAATT

>TREU927_9S_#2
ATGATAATGATGATAATGATAGGAGAGTGTTGTGAGTCTGT--
ATATACCAGTATTATAATAAGAGCAGTAATAATA
>TREU927_9S_#3
ATAATAATGATGATGATGATAGGAGAGTGTTTTGAGTGTGTGTATATACGAATATTATAATAAGAGCAGC
AGTAATA
>TREU927_9S_#4
ATAATAATGATGATAATAATAGGAGAGTGTTGTGAGTGCGTGT--
ATACGAATATTATAATAAGAGCAGTAATGATA
>TREU927_9S_#5
ATAATAATAATGATAATAATAGGAGAGTGTTGTGAGTGTGTGTATATGCGAATATTATAATAAGTGCAGT
TATGATA
>TREU927_9S_#6
ATAATAATAATAATGATAATAGGAGTGTGTTGTGAGTGTGT--
ATATACAAATATTATAATAAGAACAATAATAATA
>TREU927_9S_#7
ACAATAATAATAATAATAATAGGAGAGTGTTGTGAGTGTGTGTATATACGAATATTATAATAAGAGCAGT
AATAATA
>TREU927_9S_#8
ATGATGATAATAATAATAATAGCAGAATGTTGTGAGTGTGTGTATATACGAATATTATAATAAGAGCAGT
AATAATA
>TREU927_9S_#9
AGAATAATAATGATAATGATAGGAGAGTGTTGTGAGTGTGTGTATATACGAATATTATAATAAGAGCAGT
AATGATG
>TREU927_9S_#10
GAGCAGTAATGATGATAAGGATAAGAGAGTTGTGAGTGTGAGTATATACGAATATTATAATAAGAGCAGT
AATGATA
>TREU927_9S_#11
ATAATGATAATGATGATAGTGGGAAAGTGTTGTGAGAGTG--
TATATACGAATATTATAATAAGAGAAGTAATAATA
>TREU927_9S_#12
GTAATAATAATAATAATAATATGAGAGTGTTGTGAGTGTGTGTATATACGAATATTATAATATAAGCAGT
AATGATG
>TREU927_9S_#13
ATGATAATAATGATAATAATAGGAGAGTGCTGTGAGTCTGT--
ATATACGAATATAATAATAAGAGTAATAATAATA
>TREU927_9S_#14
ATAATGATAATAATAATAATAGTGGAGTGTTGTGAGTGTGTGTATATACGAATATTATAATAAAAGCAGT
AATAATA
>TREU927_9S_#15
ATGATAATAATGATAATAATAGGAGAGTGTTGTGAGAGTGTGCATATACGAATATTATAATAAGAGCAGT
AATAATA
>TREU927_9S_#16
ATAATAATGATAATAATAATAAGAGAGTATTGTGAGTGTA-
GTATATACAAATATTATAATAAGAGCAGTAATAATA
>TREU927_9S_#17
ATAATAATAATGATAATAATAGGAGAGTGTTGTGAGAGTGTATGTATATTCGAATATTATGTTAAGAACA
GTAATAATA
>TREU927_9S_#18
ATAATGATGATAATAATAATAGAAGAGTGTTGTGAGTGTGTGTA--

TACGAATATTATAATAAGAGCAGTAATAATA
