# Supplementary material for: A Conserved DNA Repeat Promotes Selection of a Diverse Repertoire of Trypanosoma brucei Surface Antigens from the Genomic Archive
Source: PLoS Genet. 2016 May 5;12(5):e1005994. doi: 10.1371/journal.pgen.1005994 (PMC4858185; doi:10.1371/journal.pgen.1005994)
Supplement: S1 Table — Minichromosomal DNA was isolated by gel fractionation, deep sequenced and contigs assembled. Table shows contigs containing VSG name and distance to the first upstream 70-bp repeat. (PDF) [file pgen.1005994.s005.pdf]

Contig #	Contig Length	Lister427 *VSG*#	Distance to Repeat
25	3087	643	1654
29	2138	377	429
35	4398	825	2267
40	2661	542	1032
41	3524	629	1946
43	2550	315	894
48	2376	541	891
51	3257	567	1412
53	2906	3340	987
54	2368	666	1027
55	5319	23	3177
59	3180	600	1445
61	2561	1963	827
62	3427	832	1697
63	3244	365	1672
64	2591	618	939
65	3135	663	1637
66	3747	369	1638
68	3316	507	1645
69	3601	416	1421
72	3452	622	1872
73	3958	2075	2226
78	3050	3338	1400
79	3400	620	1864
85	2705	637	1223
86	2918	717	1463
89	3379	476	1271
91	2645	575	1091
92	2817	444	1093
93	2530	646	1042
94	2470	647	775
95	3205	826	1506
		**AVERAGE**	**1421**
		**STDEV**	**537**
