# Supplementary material for: A Conserved DNA Repeat Promotes Selection of a Diverse Repertoire of Trypanosoma brucei Surface Antigens from the Genomic Archive
Source: PLoS Genet. 2016 May 5;12(5):e1005994. doi: 10.1371/journal.pgen.1005994 (PMC4858185; doi:10.1371/journal.pgen.1005994)
Supplement: S2 Table — Assigned clone numbers for each switched clone arising from DSB induction in the 70.II-ISceI line are shown alongside their population number (from 5 total), determined switching mechanism, the Lister427 number of the newly expressed VSG and its predicted location of genomic origin. (PDF) [file pgen.1005994.s006.pdf]

70.II Switched Clones	Population #	Switch Mechanism	*VSG*	Genomic Location
2_A1	1	GC	*427-631*	UD
2_A2	1	GC	*427-8*	BES14
2_A3	1	GC	*427-17*	BES13
2_A5	1	GC	*427-8*	BES14
2_A6	1	GC	*427-8*	BES14
2_A8	1	GC	*427-25*	MC
2_B1	1	GC	*427-21*	BES4
2_B10	1	GC	*427-6*	BES3
2_B11	1	GC	*427-631*	UD
2_B2	1	GC	*427-374*	MC
2_B3	1	GC	*427-8*	BES14
2_B5	1	GC	*427-6*	BES3
2_B6	1	GC	*427-11*	BES15
2_B7	1	GC	*427-8*	BES14
2_B8	1	GC	*427-3*	BES7
2_B9	1	GC	*427-24*	MC
2_C1	1	GC	*427-6*	BES3
2_C10	1	GC	*427-3*	BES7
2_C11	1	GC	*427-567*	MC
2_C3	1	GC	*427-8*	BES14
2_C5	1	GC	*427-430*	MC
2_C6	1	GC	*427-17*	BES13
2_C7	1	GC	*427-1963*	MC
2_C8	1	GC	*427-9*	BES2
2_C9	1	GC	*427-1*	UD
2_D1	1	GC	*427-3*	BES7
2_D2	1	GC	*427-3*	BES7
2_D3	1	GC	*427-365*	MC
2_D4	1	GC	*427-3*	BES7
2_D5	1	GC	*427-2057*	MC
2_D6	1	GC	*427-11*	BES15
2_E10	2	GC	*427-17*	BES13
2_E11	2	GC	*427-503*	MC
2_E12	2	GC	*427-1*	UD
2_E2	2	GC	*427-8*	BES14
2_E5	2	GC	*427-3*	BES7
2_E6	2	GC	*427-9*	BES2
2_E9	2	GC	*427-2057*	MC
2_F1	2	GC	*427-21*	BES4
2_F11	2	GC	*427-8*	BES14
2_F2	2	GC	*427-9*	BES2
2_F3	2	GC	*427-8*	BES14
2_F4	2	GC	*427-832*	MC
2_F6	2	GC	*427-3039*	UD
2_F7	2	GC	*427-12*	UD
2_G1	2	GC	*427-18*	BES5
2_G10	2	GC	*427-615*	UD
2_G11	2	GC	*427-1*	UD
2_G12	2	GC	*427-17*	BES13
2_G4	2	GC	*427-9*	BES2
2_G6	2	GC	*427-430*	MC
2_G7	2	GC	*427-3039*	UD

2_G9	2	GC	*427-476*	MC
2_H1	2	GC	*427-23*	MC
2_H5	2	GC	*427-8*	BES14
4_E1	3	GC	*427-8*	BES14
4_E10	3	GC	*427-567*	MC
4_E11	3	GC	*427-832*	MC
4_E12	3	GC	*427-3*	BES7
4_E2	3	GC	*427-567*	MC
4_E3	3	GC	*427-3039*	UD
4_E4	3	GC	*427-3039*	UD
4_E5	3	GC	*427-9*	BES2
4_E6	3	GC	*427-11*	BES15
4_E7	3	GC	*427-12*	UD
4_E8	3	GC	*427-12*	UD
4_E9	3	GC	*427-832*	MC
4_F1	3	GC	*427-21*	BES4
4_F10	3	GC	*427-17*	BES13
4_F11	3	GC	*427-631*	UD
4_F12	3	GC	*427-6*	BES3
4_F2	3	GC	*427-1963*	MC
4_F3	3	GC	*427-8*	BES14
4_F6	3	GC	*427-1*	UD
4_F7	3	GC	*427-365*	MC
4_F8	3	GC	*427-1*	UD
4_F9	3	GC	*427-631*	UD
4_G1	3	GC	*427-1*	UD
4_G2	3	GC	*427-23*	MC
4_G3	3	GC	*427-24*	MC
4_G4	3	GC	*427-9*	BES2
4_G5	3	GC	*427-476*	MC
4_G6	3	GC	*427-23*	MC
5_F1	4	GC	*427-9*	BES2
5_F10	4	GC	*427-8*	BES14
5_F11	4	GC	*427-643*	MC
5_F3	4	GC	*427-3*	BES7
5_F4	4	GC	*427-11*	BES15
5_F5	4	GC	*427-1*	UD
5_F6	4	GC	*427-3*	BES7
5_F8	4	GC	*427-615*	UD
5_F9	4	GC	*427-629*	MC
5_G10	4	GC	*427-12*	UD
5_G11	4	GC	*427-1*	UD
5_G12	4	GC	*427-11*	BES15
5_G3	4	GC	*427-8*	BES14
5_G5	4	GC	*427-24*	MC
5_G7	4	GC	*427-663*	MC
5_G9	4	GC	*427-17*	BES13
5_H2	4	GC	*427-1*	UD
5_H3	4	GC	*427-832*	MC
5_H4	4	GC	*427-3*	BES7
5_H5	4	GC	*427-9*	BES2
5_H6	4	GC	*427-17*	BES13
6_2_A1	5	GC	*427-1*	UD
6_2_A10	5	GC	*427-9*	BES2
6_2_A11	5	GC	*427-9*	BES2

6_2_A12	5	GC	*427-6*	BES3
6_2_A2	5	GC	*427-9*	BES2
6_2_A3	5	GC	*427-1*	UD
6_2_A4	5	GC	*427-12*	UD
6_2_A5	5	GC	*427-476*	MC
6_2_A6	5	GC	*427-8*	BES14
6_2_A7	5	GC	*427-8*	BES14
6_2_A8	5	GC	*427-8*	BES14
6_2_A9	5	GC	*427-417*	UD
6_2_B1	5	GC	*427-9*	BES2
6_2_B10	5	GC	*427-832*	MC
6_2_B11	5	GC	*427-3*	BES7
6_2_B12	5	GC	*427-1*	UD
6_2_B2	5	GC	*427-9*	BES2
6_2_B3	5	GC	*427-8*	BES14
6_2_B4	5	GC	*427-17*	BES13
6_2_B5	5	GC	*427-1*	UD
6_2_B6	5	GC	*427-567*	MC
6_2_B7	5	GC	*427-11*	BES15
6_2_B8	5	GC	*427-3039*	UD
6_2_B9	5	GC	*427-17*	BES13
6_2_C1	5	GC	*427-3*	BES7
6_2_C2	5	GC	*427-6*	BES3
6_2_C3	5	GC	*427-1*	UD
6_2_C4	5	GC	*427-1963*	MC
6_2_C5	5	GC	*427-8*	BES14
