# Supplementary material for: A Conserved DNA Repeat Promotes Selection of a Diverse Repertoire of Trypanosoma brucei Surface Antigens from the Genomic Archive
Source: PLoS Genet. 2016 May 5;12(5):e1005994. doi: 10.1371/journal.pgen.1005994 (PMC4858185; doi:10.1371/journal.pgen.1005994)
Supplement: S3 Table — Assigned clone numbers for each switched clone arising from DSB induction in the 70.I-ISceI line are shown alongside their population number (from 5 total), determined switching mechanism, the Lister427 number of the newly expressed VSG and its predicted location of genomic origin. (PDF) [file pgen.1005994.s007.pdf]

70.I Switched Clones	Population #	Switch Mechanism	*VSG*	Genomic Location
4_A1	1	GC	*427-3*	BES7
4_A10	1	GC	*427-9*	BES2
4_A11	1	GC	*427-9*	BES2
4_A2	1	GC	*427-3*	BES7
4_A3	1	GC	*427-17*	BES13
4_A4	1	GC	*427-9*	BES2
4_A5	1	GC	*427-3*	BES7
4_A6	1	GC	*427-9*	BES2
4_A7	1	GC	*427-9*	BES2
4_A8	1	GC	*427-17*	BES13
4_B1	1	GC	*427-8*	BES14
4_B10	1	GC	*427-3*	BES7
4_B11	1	GC	*427-8*	BES14
4_B2	1	GC	*427-17*	BES13
4_B3	1	GC	*427-8*	BES14
4_B4	1	GC	*427-8*	BES14
4_B5	1	GC	*427-8*	BES14
4_B6	1	GC	*427-6*	BES3
4_B7	1	GC	*427-11*	BES15
4_B8	1	GC	*427-9*	BES2
4_B9	1	GC	*427-3*	BES7
4_C1	1	GC	*427-8*	BES14
4_C2	1	GC	*427-3*	BES7
4_C4	1	GC	*427-3*	BES7
4_C5	1	GC	*427-8*	BES14
4_C6	1	GC	*427-8*	BES14
5_A1	2	GC	*427-8*	BES14
5_A10	2	GC	*427-3*	BES7
5_A11	2	GC	*427-3*	BES7
5_A2	2	GC	*427-3*	BES7
5_A3	2	GC	*427-8*	BES14
5_A4	2	GC	*427-11*	BES15
5_A5	2	GC	*427-8*	BES14
5_A6	2	GC	*427-3*	BES7
5_A7	2	GC	*427-9*	BES2
5_A9	2	GC	*427-3*	BES7
5_B1	2	GC	*427-9*	BES2
5_B10	2	GC	*427-8*	BES14
5_B11	2	GC	*427-17*	BES13
5_B12	2	GC	*427-3*	BES7
5_B2	2	GC	*427-3*	BES7
5_B3	2	GC	*427-3*	BES7
5_B5	2	GC	*427-8*	BES14
5_B6	2	GC	*427-3*	BES7
5_B8	2	GC	*427-8*	BES14
5_B9	2	GC	*427-8*	BES14
5_C1	2	GC	*427-9*	BES2

5_C11	3	GC	*427-8*	BES14
5_C12	3	GC	*427-8*	BES14
5_C2	2	GC	*427-9*	BES2
5_C4	2	GC	*427-8*	BES14
5_C5	2	GC	*427-8*	BES14
5_C6	2	GC	*427-21*	BES4
5_C8	3	GC	*427-8*	BES14
5_C9	3	GC	*427-3*	BES7
5_D10	3	GC	*427-11*	BES15
5_D11	3	GC	*427-3*	BES7
5_D12	3	GC	*427-11*	BES15
5_D2	3	GC	*427-8*	BES14
5_D4	3	GC	*427-17*	BES13
5_D6	3	GC	*427-17*	BES13
5_D7	3	GC	*427-17*	BES13
5_D8	3	GC	*427-3*	BES7
5_D9	3	GC	*427-3*	BES7
5_E1	3	GC	*427-3*	BES7
5_E10	3	GC	*427-11*	BES15
5_E11	3	GC	*427-3*	BES7
5_E12	3	GC	*427-3*	BES7
5_E2	3	GC	*427-3*	BES7
5_E4	3	GC	*427-3*	BES7
5_E5	3	GC	*427-3*	BES7
5_E6	3	GC	*427-3*	BES7
5_E8	3	GC	*427-9*	BES2
5_E9	3	GC	*427-9*	BES2
6_1_A1	4	GC	*427-3*	BES7
6_1_A11	4	GC	*427-9*	BES2
6_1_A12	4	GC	*427-17*	BES13
6_1_A2	4	GC	*427-9*	BES2
6_1_A3	4	GC	*427-3*	BES7
6_1_A4	4	GC	*427-8*	BES14
6_1_A5	4	GC	*427-11*	BES15
6_1_A6	4	GC	*427-8*	BES14
6_1_A7	4	GC	*427-9*	BES2
6_1_A8	4	GC	*427-17*	BES13
6_1_A9	4	GC	*427-25*	MC
6_1_B1	4	GC	*427-3*	BES7
6_1_B10	4	GC	*427-17*	BES13
6_1_B11	4	GC	*427-8*	BES14
6_1_B12	4	GC	*427-17*	BES13
6_1_B3	4	GC	*427-3*	BES7
6_1_B4	4	GC	*427-9*	BES2
6_1_B5	4	GC	*427-9*	BES2
6_1_B8	4	GC	*427-9*	BES2
6_1_B9	4	GC	*427-9*	BES2
6_1_C1	4	GC	*427-11*	BES15
6_1_C2	4	GC	*427-9*	BES2

6_1_C3	4	GC	*427-9*	BES2
6_1_C4	4	GC	*427-8*	BES14
6_1_C5	4	GC	*427-12*	UD
6_1_C6	4	GC	*427-9*	BES2
6_1_E1	5	GC	*427-17*	BES13
6_1_E10	5	GC	*427-3*	BES7
6_1_E11	5	GC	*427-8*	BES14
6_1_E12	5	GC	*427-17*	BES13
6_1_E2	5	GC	*427-17*	BES13
6_1_E3	5	GC	*427-9*	BES2
6_1_E4	5	GC	*427-3*	BES7
6_1_E5	5	GC	*427-11*	BES15
6_1_E6	5	GC	*427-17*	BES13
6_1_E7	5	GC	*427-6*	BES3
6_1_E8	5	GC	*427-17*	BES13
6_1_E9	5	GC	*427-3*	BES7
6_1_F10	5	GC	*427-8*	BES14
6_1_F11	5	GC	*427-3*	BES7
6_1_F12	5	GC	*427-9*	BES2
6_1_F2	5	GC	*427-8*	BES14
6_1_F3	5	GC	*427-23*	MC
6_1_F4	5	GC	*427-11*	BES15
6_1_F5	5	GC	*427-631*	UD
6_1_F6	5	GC	*427-17*	BES13
6_1_F7	5	GC	*427-17*	BES13
6_1_F8	5	GC	*427-17*	BES13
6_1_F9	5	GC	*427-21*	BES4
6_1_G1	5	GC	*427-17*	BES13
6_1_G3	5	GC	*427-11*	BES15
6_1_G5	5	GC	*427-17*	BES13
6_1_G6	5	GC	*427-3*	BES7
