# Supplementary material for: A Conserved DNA Repeat Promotes Selection of a Diverse Repertoire of Trypanosoma brucei Surface Antigens from the Genomic Archive
Source: PLoS Genet. 2016 May 5;12(5):e1005994. doi: 10.1371/journal.pgen.1005994 (PMC4858185; doi:10.1371/journal.pgen.1005994)
Supplement: S4 Table — Assigned clone numbers for each switched clone arising from DSB induction in the 70.II-ISceI line are shown alongside their population number (from 5 total), determined switching mechanism, the Lister427 number of the newly expressed VSG and its predicted location of genomic origin. (PDF) [file pgen.1005994.s008.pdf]

Δ70 Switched Clones	Population #	Switch Mechanism	*VSG*	Genomic Location
1_A1	1	GC	*427-8*	BES14
1_A2	1	GC	*427-9*	BES2
1_A3	1	GC	*427-9*	BES2
1_A4	1	GC	*427-3*	BES7
1_A5	1	GC	*427-3*	BES7
1_A6	1	GC	*427-21*	BES4
1_A7	1	GC	*427-3*	BES7
1_A8	1	GC	*427-11*	BES15
1_A9	1	GC	*427-8*	BES14
1_B10	1	GC	*427-8*	BES14
1_B2	1	GC	*427-8*	BES14
1_B3	1	GC	*427-9*	BES2
1_B4	1	GC	*427-9*	BES2
1_B6	1	GC	*427-8*	BES14
1_B7	1	GC	*427-9*	BES2
1_B9	1	GC	*427-17*	BES13
1_C4	1	GC	*427-11*	BES15
1_C5	1	GC	*427-3*	BES7
1_E1	2	GC	*427-18*	BES5
1_E10	2	GC	*427-8*	BES14
1_E3	2	GC	*427-9*	BES2
1_E4	2	GC	*427-11*	BES15
1_E5	2	GC	*427-9*	BES2
1_E6	2	GC	*427-9*	BES2
1_E7	2	GC	*427-8*	BES14
1_E9	2	GC	*427-17*	BES13
1_F1	2	GC	*427-17*	BES13
1_F10	2	GC	*427-17*	BES13
1_F11	2	GC	*427-9*	BES2
1_F12	2	GC	*427-9*	BES2
1_F2	2	GC	*427-17*	BES13
1_F3	2	GC	*427-11*	BES15
1_F4	2	GC	*427-17*	BES13
1_F5	2	GC	*427-17*	BES13
1_F7	2	GC	*427-17*	BES13
1_F8	2	GC	*427-11*	BES15
1_F9	2	GC	*427-9*	BES2
1_G1	2	GC	*427-17*	BES13
1_G2	2	GC	*427-17*	BES13
1_G3	2	GC	*427-17*	BES13
1_G5	2	GC	*427-17*	BES13
1_G6	2	GC	*427-17*	BES13
1_G7	2	GC	*427-8*	BES14
3_A1	3	GC	*427-6*	BES3
3_A11	3	GC	*427-3*	BES7
3_A12	3	GC	*427-9*	BES2
3_A2	3	GC	*427-3*	BES7
3_A3	3	GC	*427-3*	BES7
3_A5	3	GC	*427-11*	BES15
3_A7	3	GC	*427-3*	BES7
3_A8	3	GC	*427-11*	BES15
3_A9	3	GC	*427-3*	BES7

3_B1	3	GC	*427-18*	BES5
3_B10	4	GC	*427-3*	BES7
3_B12	4	GC	*427-11*	BES15
3_B2	3	GC	*427-11*	BES15
3_B3	3	GC	*427-3*	BES7
3_B5	3	GC	*427-3*	BES7
3_B6	3	GC	*427-11*	BES15
3_B7	4	GC	*427-3*	BES7
3_B8	4	GC	*427-11*	BES15
3_B9	4	GC	*427-8*	BES14
3_C1	4	GC	*427-9*	BES2
3_C10	4	GC	*427-17*	BES13
3_C11	4	GC	*427-9*	BES2
3_C2	4	GC	*427-11*	BES15
3_C3	4	GC	*427-17*	BES13
3_C4	4	GC	*427-3*	BES7
3_C5	4	GC	*427-8*	BES14
3_C6	4	GC	*427-17*	BES13
3_C9	4	GC	*427-3*	BES7
3_D1	4	GC	*427-3*	BES7
3_D10	4	GC	*427-3*	BES7
3_D11	4	GC	*427-9*	BES2
3_D2	4	GC	*427-3*	BES7
3_D3	4	GC	*427-3*	BES7
3_D5	4	GC	*427-17*	BES13
3_D7	4	GC	*427-3*	BES7
3_D8	4	GC	*427-6*	BES3
3_E1	4	GC	*427-11*	BES15
3_E10	4	GC	*427-17*	BES13
3_E11	4	GC	*427-17*	BES13
3_E12	4	GC	*427-3*	BES7
3_E2	4	GC	*427-17*	BES13
3_E3	4	GC	*427-3*	BES7
3_E4	4	GC	*427-3*	BES7
3_E5	4	GC	*427-8*	BES14
3_E8	4	GC	*427-8*	BES14
3_E9	4	GC	*427-3*	BES7
6_2_E1	5	GC	*427-3*	BES7
6_2_E10	5	GC	*427-8*	BES14
6_2_E11	5	GC	*427-8*	BES14
6_2_E12	5	GC	*427-8*	BES14
6_2_E2	5	GC	*427-11*	BES15
6_2_E3	5	GC	*427-17*	BES13
6_2_E4	5	GC	*427-11*	BES15
6_2_E5	5	GC	*427-3*	BES7
6_2_E6	5	GC	*427-9*	BES2
6_2_E7	5	GC	*427-9*	BES2
6_2_E8	5	GC	*427-3*	BES7
6_2_E9	5	GC	*427-11*	BES15
6_2_F1	5	GC	*427-9*	BES2
6_2_F10	5	GC	*427-9*	BES2
6_2_F11	5	GC	*427-8*	BES14
6_2_F12	5	GC	*427-8*	BES14
6_2_F2	5	GC	*427-11*	BES15
6_2_F3	5	GC	*427-18*	BES5

6_2_F4	5	GC	*427-9*	BES2
6_2_F5	5	GC	*427-17*	BES13
6_2_F6	5	GC	*427-11*	BES15
6_2_F7	5	GC	*427-9*	BES2
6_2_F8	5	GC	*427-9*	BES2
6_2_F9	5	GC	*427-9*	BES2
6_2_G1	5	GC	*427-3*	BES7
6_2_G2	5	GC	*427-9*	BES2
6_2_G3	5	GC	*427-8*	BES14
6_2_G4	5	GC	*427-9*	BES2
6_2_G5	5	GC	*427-3*	BES7
6_2_G6	5	GC	*427-9*	BES2
