# Supplementary material for: A Conserved DNA Repeat Promotes Selection of a Diverse Repertoire of Trypanosoma brucei Surface Antigens from the Genomic Archive
Source: PLoS Genet. 2016 May 5;12(5):e1005994. doi: 10.1371/journal.pgen.1005994 (PMC4858185; doi:10.1371/journal.pgen.1005994)
Supplement: S5 Table — VSG gene numbers (Lister427) for all VSGs selected from these experiments are shown along site their corresponding genomic locations and their proportion within the population as determined by VSG-seq for three replicates of each line analyzed (Δ70-ISceI, Dimer and Dimer_Rv). (PDF) [file pgen.1005994.s009.pdf]

VSG-seq data from minimal repeat introductions

Lister427 VSG	Genomic Location	Δ70.1	Δ70.2	Δ70.3	Dimer.1	Dimer.2	Dimer.3	Dimer_Rv.1	Dimer_Rv.2	Dimer_Rv.3
427-17var	BES	34.752	32.573	26.054	22.406	22.376	19.322	24.194	27.162	24.058
427-3	BES	19.772	20.794	28.207	10.023	13.791	14.816	27.848	21.828	24.417
427-8	BES	12.571	13.389	16.999	29.894	25.909	28.281	17.361	17.808	16.798
427-11	BES	15.516	14.159	11.977	20.480	21.783	18.646	16.872	18.750	18.043
427-9	BES	6.472	6.158	5.038	5.859	5.834	7.258	5.098	4.809	4.305
427-18	BES	3.578	3.810	5.203	3.088	2.507	2.067	2.309	2.544	2.746
427-13var	BES	1.020	1.099	1.036	0.976	1.775	0.989	2.468	2.762	4.165
427-21	BES	1.177	1.387	1.003	2.585	2.203	2.483	1.154	2.008	1.590
427-6	BES	0.789	0.761	0.422	1.471	1.198	2.229	1.031	0.771	1.112
427-631var	UD	0.927	0.783	0.670	0.608	0.496	0.778	0.367	0.315	0.565
427-775var	UD	0.241	0.264	0.144	0.158	0.162	0.220	0.852	0.695	0.985
427-15	BES	0.518	0.439	0.483	0.179	0.087	0.143	0.076	0.081	0.237
427-653	MES	0.460	0.273	0.265	0.064	0.050	0.113	0.032	0.057	0.126
427-531	MES	0.169	0.171	0.192	0.032	0.023	0.059	0.050	0.035	0.091
427-1	UD	0.000	0.000	0.000	0.135	0.067	0.112	0.119	0.104	0.112
427-663	MC	0.000	0.000	0.000	0.159	0.127	0.327	0.000	0.000	0.000
427-417	UD	0.000	0.000	0.000	0.252	0.134	0.201	0.000	0.000	0.000
427-567	MC	0.000	0.000	0.000	0.060	0.223	0.156	0.000	0.000	0.000
427-22	MC	0.000	0.000	0.000	0.120	0.081	0.192	0.000	0.000	0.000
427-1963	MC	0.000	0.000	0.000	0.167	0.077	0.142	0.000	0.000	0.000
427-615	UD	0.011	0.000	0.000	0.115	0.026	0.108	0.000	0.124	0.000
427-25	MC	0.000	0.019	0.000	0.076	0.071	0.130	0.022	0.000	0.026
427-636	UD	0.043	0.035	0.044	0.013	0.014	0.032	0.020	0.036	0.091
427-629	MC	0.000	0.000	0.000	0.020	0.105	0.075	0.013	0.000	0.000
427-2057var1	MC	0.000	0.000	0.000	0.077	0.037	0.067	0.000	0.000	0.016
427-12	UD	0.000	0.000	0.000	0.081	0.052	0.040	0.000	0.000	0.000
427-3039var	UD	0.000	0.000	0.000	0.050	0.050	0.056	0.000	0.000	0.000
427-416	MC	0.000	0.000	0.000	0.063	0.040	0.052	0.000	0.000	0.000
427-1591var	UD	0.015	0.014	0.016	0.015	0.010	0.033	0.011	0.000	0.015
427-365	MC	0.000	0.054	0.018	0.000	0.000	0.018	0.014	0.000	0.000
427-322	UD	0.000	0.000	0.000	0.021	0.011	0.053	0.000	0.000	0.011
427-23	MC	0.000	0.026	0.000	0.000	0.015	0.042	0.000	0.000	0.000
427-476	MC	0.000	0.000	0.000	0.014	0.000	0.041	0.000	0.000	0.000
427-14var	BES	0.000	0.012	0.000	0.011	0.000	0.019	0.000	0.000	0.012
427-430	MC	0.000	0.000	0.000	0.015	0.000	0.036	0.000	0.000	0.000
427-2057var2	MC	0.000	0.000	0.000	0.016	0.000	0.010	0.012	0.000	0.012
427-1123	UD	0.000	0.000	0.000	0.010	0.013	0.021	0.000	0.000	0.000
427-510	UD	0.000	0.000	0.000	0.000	0.000	0.037	0.000	0.000	0.000
427-374	MC	0.000	0.000	0.000	0.000	0.000	0.026	0.000	0.000	0.000
427-1387var	UD	0.000	0.000	0.000	0.000	0.000	0.024	0.000	0.000	0.000
427-24var	MC	0.000	0.000	0.000	0.000	0.000	0.013	0.000	0.000	0.000
427-637	MC	0.000	0.000	0.000	0.000	0.000	0.000	0.000	0.000	0.011
427-503var	MC	0.000	0.000	0.000	0.000	0.000	0.000	0.000	0.000	0.010
427-1var	UD	0.000	0.000	0.000	0.000	0.000	0.000	0.010	0.000	0.000

"var" - indicates that it the identified sequence is a minor variation of the published sequence for the corresponding VSG#.
