# Supplementary material for: A Conserved DNA Repeat Promotes Selection of a Diverse Repertoire of Trypanosoma brucei Surface Antigens from the Genomic Archive
Source: PLoS Genet. 2016 May 5;12(5):e1005994. doi: 10.1371/journal.pgen.1005994 (PMC4858185; doi:10.1371/journal.pgen.1005994)
Supplement: S1 Text — Red Nucleotides Show BES1 homology. Long oligos used to make repeats are shown as Monomer, Dimer, or Mut_Dimer. Only forward oligo shown, KpnI sites were used to clong repeat fragments into Δ70-ISceI landscape. (PDF) [file pgen.1005994.s010.pdf]

70.II-ISceI Construction
"70.IIRS_GHMFw1":
AATAGGAGAGTGTTGTGAGTGTGTGCTTACCAATATTATAATAATGATAGTAACGACCAAT
AGGGATAACAGGGTAATACCGGTATAACTTCGTATAGCATACATTATACGAAGTTATCTCG
"70.IIRS_GHMRv1":
CAAATCCATTATACTCACGATTACTCTCATTGCACACATACCATTGTCTTAACTGCATTTAT
TTATGGTTTGTATTCGTCATAACTTCGTATAATGTATGCTATACGAAGTTATCCTCTAATCC
TTTCACTCTTCCG

70.I-ISceI Construction
"70.IRS_Fw1":
ATATGCGGCCGCATGACAGCAGTTTTAGACACTAAAGGCGGAGCCCAAATAGAGGCACTT
ACAACTTTAGAAAAAATTAAATTAGAGTAGGGATAACAGGGTAATACCGGTATAACTTCGT
ATAGCATACATTATACGAAGTTATCTCG
"70.IRS_Rv1":
ATATGCGGCCGCATGCAGCTGCGCATATACAATATCACTTTCTATATTTGTCTTTCTGCGT
TTCCTTTAATATATTTCTAAGTGCGCTCCAACTTACATAACTTCGTATAATGTATGCTATAC
GAAGTTATCCTCTAATCCTTTCACTCTTCCG

Δ70-ISceI Construction: Flanking regions were sewn to PURO cassette by long-flanking homology PCR. Oligos for amplification of the 5' and 3' flanking homology regions are shown. PCR product resulted in ~1.5Kb of homology to BES1 on each side of cassettee.
5'-Flanking homology
"NotI ESAG1 UQ Fw":
ATATGCGGCCGCATGGAAGAGCAAACTGATAGGTTGGAAAAGC
"-70 P2 NEW":
CCCTCGAGATAACTTCGTATAATGTATGCTATACGAAGTTATACCGGTATTACCCTGTTAT
CCCTACTTTTAACCTTGTGCCCGACCTTTACAGTC
3'-Flanking homology
"-70 P2 #3 (KpnI)":
CCCTCGAGATAACTTCGTATAATGTATGCTATACGAAGTTATTATTACCCTGTTATCCCTAG
GTACCCTTTTAACCTTGTGCCCGACCTTTACAGTC
"VSG221 5'NotI Rv1":
ATATGCGGCCGCATGGAAGAACTTGGGCTAGGACCAAGACG

Monomer Oligo
6-77-Fw
ATAGGTACCATAATAATAATAATAATAATAATAATAGGAGAGTGTTGTGAGTGTGTGTATAT
ACGAATATTATAATAAGAGCAGTAATAATAATAATAGGTACCATA
Dimer Oligo
2x-6-77-Fw
ATAGGTACCATAATAATAATAATAATAATAATAATAGGAGAGTGTTGTGAGTGTGTGTATAT
ACGAATATTATAATAAGAGCAGTAATAATAATAATAATAATAATAATAATAATAATAGGAGA
GTGTTGTGAGTGTGTGTATATACGAATATTATAATAAGAGCAGTAATAATAATAATAGGTAC
CATA
Mut_Dimer Oligo
2x-6-77mut_Fw
ATAGGTACCATAATAATAATAATAATAATAATAATAAAAAAATATTATAAATATATATATATAT
AAATATTATAATAAAAATAATAATAATAATAATAATAATAATAATAATAATAATAAAAAAATAT
TATAAATATATATATATATAAATATTATAATAAAAATAATAATAATAATAATAGGTACCATA
